# Jahn–Teller Distortions and Phase Transitions in LiNiO_2_: Insights from Ab Initio Molecular Dynamics and Variable-Temperature X-ray Diffraction

**DOI:** 10.1021/acs.chemmater.3c02413

**Published:** 2024-02-20

**Authors:** Annalena R. Genreith-Schriever, Alexandra Alexiu, George S. Phillips, Chloe S. Coates, Liam A. V. Nagle-Cocco, Joshua D. Bocarsly, Farheen N. Sayed, Siân E. Dutton, Clare P. Grey

**Affiliations:** † Yusuf Hamied Department of Chemistry, University of Cambridge, Cambridge CB2 1EW, U.K.; ‡ Cavendish Laboratory, University of Cambridge, Cambridge CB3 0HE, U.K.; § Department of Chemistry, University of Houston, Houston, Texas 77204-5003, United States; ∥ The Faraday Institution, Harwell Science and Innovation Campus, Didcot OX11 0RA, U.K.

## Abstract

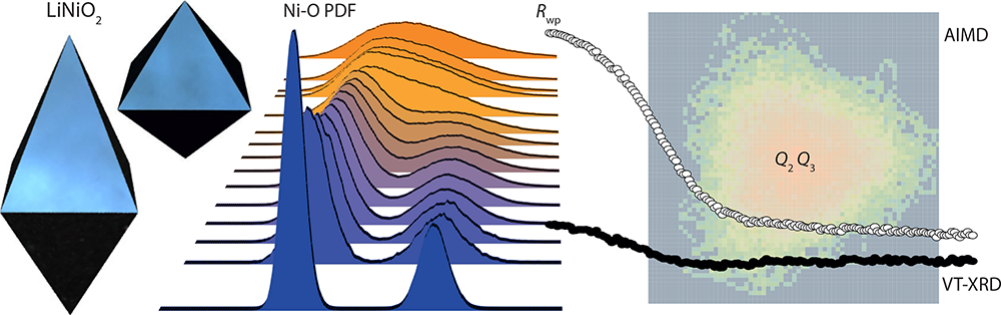

The atomistic structure of lithium nickelate (LiNiO_2_), the parent compound of Ni-rich layered oxide cathodes for Li-ion batteries, continues to elude a comprehensive understanding. The common consensus is that the material exhibits local Jahn–Teller distortions that dynamically reorient, resulting in a time-averaged undistorted *R*3̅*m* structure. Through a combination of ab initio molecular dynamics (AIMD) simulations and variable-temperature X-ray diffraction (VT-XRD), we explore Jahn–Teller distortions in LiNiO_2_ as a function of temperature. Static Jahn–Teller distortions are observed at low temperatures (*T* < 250 K) via AIMD simulations, followed by a broad phase transition that occurs between 250 and 350 K, leading to a highly dynamic, displacive phase at high temperatures (*T* > 350 K), which does not show the four short and two long bonds characteristic of local Jahn–Teller distortions. These transitions are followed in the AIMD simulations via abrupt changes in the calculated pair distribution function and the bond-length distortion index and in X-ray diffraction via the monoclinic lattice parameter ratio, *a*_mon_/*b*_mon_, and δ angle, the fit quality of an *R*3̅*m*-based structural refinement, and a peak sharpening of the diffraction peaks on heating, consistent with the loss of distorted domains. Between 250 and 350 K, a mixed-phase regime is found via the AIMD simulations where distorted and undistorted domains coexist. The repeated change between the distorted and undistorted states in this mixed-phase regime allows the Jahn–Teller long axes to change direction. These pseudorotations of the Ni–O long axes are a side effect of the onset of the displacive phase transition. Antisite defects, involving Li ions in the Ni layer and Ni ions in the Li layer, are found to pin the undistorted domains at low temperatures, impeding cooperative ordering at a longer length scale.

## Introduction

Ni-rich-layered oxide cathodes play a pivotal role in attempts to reduce Co contents and provide more sustainable cathode materials for Li-ion batteries. Their complexity of structural and electronic properties, however, renders the tailored design of Ni-rich cathodes challenging. The parent compound lithium nickelate LiNiO_2_ (LNO) has itself become the subject of much debate.^1−4^ In part, this is because the role that Jahn–Teller (JT) distortions of the formally Ni^3+^ (*d*^7^) ion play in controlling both short- and long-range structure is unclear. Given the potential interplay of structural distortions, electronic transport, and Li-ion conductivity, a comprehensive understanding of the nature of structural distortions in the pristine material is critical.

**Figure 1:**
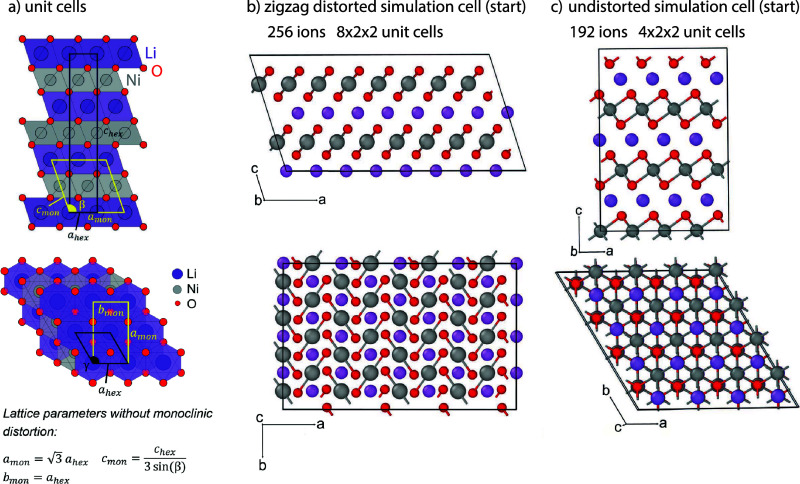
LiNiO_2_unit cells and simulation cells used in this study. (a) Structural relation between the rhombohedral unit cell (black lines) and monoclinic unit cell (yellow lines), with the lattice parameters marked. β is the cell angle of the monoclinic cell. (b) Zigzag distorted monoclinic simulation cell (starting structure). (c) Undistorted rhombohedral simulation cell (starting structure). Ni ions are shown in gray, O ions in red, and Li ions in purple.

The X-ray diffraction (XRD) and neutron diffraction patterns of LNO at room temperature can be well described by an overall undistorted, rhombohedral structure *R*3̅*m*^5^^,^^6^ (Figure 1a, see the Supporting Information), while an extended X-ray absorption fine structure (EXAFS) analysis suggests that the symmetry of the local Ni–O bonding environment is broken,^7^ typical of local Jahn–Teller distortions.

When the orientation of the Jahn–Teller distortions of neighboring NiO_6_ octahedra is strongly correlated, as in the case of the closely related NaNiO_2_, the distortions lead to a macroscopic lowering of the symmetry of the crystal structure from *R*3̅*m* to *C*2/*m*.^8^ The absence of a macroscopic change of symmetry in LNO suggests that Jahn–Teller distortions are absent or, if present, are not cooperative to a sufficient degree to drive long-range ordering. The general consensus is that the material is locally distorted, but the distortions reorient dynamically,^9^ i.e., the O–Ni–O long bond axis undergoes pseudorotations, averaging to the overall *R*3̅*m* structure. Based on density functional theory (DFT) calculations, Radin and Van Der Ven^10^ predicted that the ground state of LNO at zero Kelvin was Jahn–Teller distorted with a zigzag arrangement of the long Ni–O bonds (Figure 1b) (a collinear arrangement being 12 meV per formula unit higher in energy). On the basis of neutron diffraction, Chung et al.^6^ have proposed a trimer arrangement of distortions where three long Ni–O bonds point toward each Ni ion. The trimer ordering, however, has since been shown to be energetically unfavorable.^10^ Sicolo et al.^9^ proposed the occurrence of pseudorotations based on their ab initio molecular dynamics (AIMD) simulations.^9^ Middlemiss et al.^11^ analyzed the experimental solid-state nuclear magnetic resonance spectra of LNO and LiNi_*x*_Co_1–*x*_O_2_, calculating hyperfine shifts for static and dynamic Jahn–Teller distortions, and their results supported dynamic Jahn–Teller distortions. The dynamic behavior is expected to be frozen in at low temperatures,^1^ and neutron diffraction experiments performed at 10 K can indeed be fitted well with a distorted *C*2/*m* structure confirming the drive of LNO to assume a distorted state at low temperatures.^6^

Radin et al. conceptually explored the nature of phase transitions in materials with structural distortions. On heating, a Jahn–Teller distorted low-temperature phase can undergo either an order–disorder or a displacive phase transition to a dynamically stabilized high-temperature phase.^1^ In the order–disorder case, the local symmetry of the high-temperature phase is identical to the symmetry of the low-temperature phase, but there is no long-range order. The octahedra are still Jahn–Teller distorted locally (tetragonally elongated in the case of LiNiO_2_) at any snapshot in time but reorient dynamically, resulting in an average undistorted structure. In the case of a displacive transition, the octahedra in the high-temperature phase may be distorted locally but the distortions do not necessarily correspond to those expected for an idealized Jahn–Teller distortion; they follow a unimodal distribution with a maximum corresponding to the undistorted high-symmetry structure; i.e., they spend most time in, or close to, undistorted configurations.

As a result, the local symmetry of the high-temperature phase differs from the symmetry of the distorted low-temperature phase and averages to the undistorted parent structure. The terms “order/disorder” and “displacive” traditionally denote the type of phase transition. To introduce equally clear and brief terms to label the high-temperature phases, Radin et al. expanded the use of the terms “order/disorder” and “displacive” to describe the high-temperature phases themselves (e.g., the “displacive high-temperature phase”).^1^ We will follow their notation and also differentiate between the high-temperature phases by referring to them as distorted (following an order/disorder transition the local JT distortions are maintained) versus undistorted (following a displacive transition, the octahedra have the greatest probability of being in an undistorted state). Radin et al. predicted that LNO would have an order–disorder high-temperature phase based on an anharmonic vibrational model.^1^ This is in agreement with the general notion of dynamic Jahn–Teller distortions in LNO and order–disorder transitions observed in other Jahn–Teller distorted oxides.^12−14^ We here employ a combination of AIMD simulations and variable temperature (VT)-XRD to shed light on the nature and dynamics of the Jahn–Teller distortions in LiNiO_2_ as a function of temperature. We start by exploring the effect of temperature via AIMD on the stoichiometric structure and then investigate the impact of antisite defects. We then analyze the XRD data to explore whether the average structural information provided by a Rietveld analysis exhibits any signatures of the predicted phase behavior. We observe the direction of the Jahn–Teller axes changing dynamically around room temperature in our AIMD simulations but suggest that this does not stem from an order/disorder transition but the onset of the displacive transition, specifically from the coexistence of distorted and undistorted domains in the mixed-phase regime. Our VT-XRD diffractograms suggest the formation of domains with monoclinic character at low temperatures and the reversible loss of the distortions and domain structure around 300 K.

## Experimental Methods

### Variable-Temperature Synchrotron XRD

High-resolution X-ray diffraction was carried out on the I11 beamline at the Diamond Light Source synchrotron, UK. Polycrystalline LNO powder was obtained from BASF. The sample was packed into a 0.5-mm external diameter quartz capillary (Capillary Tube Supplies Ltd.) and sealed with epoxy under an argon environment. XRD patterns were measured by using a position-sensitive detector (PSD) and a beam energy of 15 keV (∼0.827 Å).

Following an initial room temperature scan, the sample was cooled to 100 K by using the cryostat. Continuous 5 s PSD scans were measured during heating at 6 K/min up to 500 K. Further measurements were taken during cooling back to 100 K at 6 K/min to check the reversibility of observations.

Rietveld refinements were performed using TOPAS Academic (ver. 6.0). First, the degree of antisite mixing in the LNO sample was determined from refinement of the initial room temperature XRD pattern. The Li/Ni occupancies on both the Li and Ni sites were refined to estimate 3.8(6)% Li on the Ni site and 3(1)% Ni on the Li site. These values were fixed for subsequent variable temperature analysis.

To fit a crystal structure to each diffraction pattern during the variable temperature measurements, we performed a sequential Rietveld refinement. In this process, an initial structure is provided to act as the “seed”, and the program fits the XRD pattern for each temperature in turn using the previous refined structural parameters as a new starting point. The sequential refinement was repeated using rhombohedral (*R*3̅*m*) and monoclinic (*P*2_1_/*c*) symmetries (see Supporting Information for a selection of Rietveld refinements for each).

## Theoretical Calculations

### AIMD Simulations and DFT Calculations

AIMD simulations and static DFT calculations were performed according to the Generalized Gradient Approximation (GGA) proposed by Perdew et al.,^15^ and the projector augmented wave (PAW) method,^16^ as implemented in the Vienna Ab Initio Simulation Package (VASP).^17^^,^^18^ The electronic wave functions were expanded with a basis set of plane waves with kinetic energies of up to 500 eV. Supercells with 192–256 ions and a 2 × 2 × 2 Monkhorst–Pack *k*-point mesh^19^ were used. For the structural analysis of the cell with the distorted starting structure after longer equilibration times, simulations were performed at the Γ point and checked against the 2 × 2 × 2 calculations for consistency. The convergence criteria for the electronic and ionic relaxations were set to 10^–6^ eV and 1 × 10^–3^ eV/Å, respectively.

For Ni, the 4*s*^2^3*d*^8^ electrons were treated as valence electrons. To account for the strongly correlated *d* electrons, a rotationally invariant Hubbard *U* parameter was used.^20^ The electronic density of states was calculated at varying *U*_eff_ in the range from 0 to 10 eV and compared with the density of states obtained with the screened hybrid functional proposed by Heyd et al., (HSE06)^21^ with 25% Fock exchange. The best agreement was achieved at *U*_eff_ = 6 eV, in agreement with findings by Das et al.,^4^ which was used for all AIMD and DFT calculations. For oxygen, the 2*s*^2^2*p*^4^ electrons were considered to be valence electrons.

AIMD simulations were performed for the isothermal–isobaric ensemble (*NpT*, constant pressure, particle number, and temperature) at zero pressure. A Langevin thermostat was used with friction coefficients set to zero to minimize impact on the lattice vibrations. Time-averaged pair distribution functions were evaluated with the OVITO software package.^100^

Ionic charges were analyzed based on a Wannier projection as described in ref (2).

### Analysis of Van Vleck Distortion Modes

The distortions of the NiO_6_ octahedra were quantified by analyzing the van Vleck distortion modes,^22^ irreducible representations of the possible distortions of the O ions from their ideal octahedral positions. Of the 18 modes, two modes (by convention referred to as *Q*_2_ and *Q*_3_) have the same symmetry as *e*_g_ orbitals, which is useful because distortions stemming from the Jahn–Teller effect will have the same symmetry as the degenerate orbitals causing it. *Q*_2_ is a planar rhombic distortion, and *Q*_3_ is a tetragonal distortion. It is common to calculate the two parameters for a Jahn–Teller-distorted octahedron and calculate its position within an *E*_g_(*Q*_2_,*Q*_3_) phase space.^23^^–^^25^ The magnitude of the distortion ρ_0_ can be calculated as  and the angle ϕ of this distortion from being of purely *Q*_3_ character as .

For octahedra of purely *Q*_3_ character, which is the case for LiNiO_2_, a rotation of Δϕ = 120° indicates a switching of the tetragonally elongated axis. The van Vleck modes in this study were calculated using the Python package VanVleckCalculator,^24,26^ assuming orthogonal axes.

## Results

### Zero Kelvin Calculations

In line with previous DFT studies,^10,27−29^ in our DFT calculations at 0 K we find that the cooperatively distorted structure with a zigzag orientation of the Jahn–Teller long axes (Figure 1b) is the most favorable at 0 K followed closely by the collinear distorted structure (6 meV per O higher in energy than the zigzag distorted structure) and the undistorted structure (Figure 1c).

### Temperature-Dependent Phases

**Figure 2:**
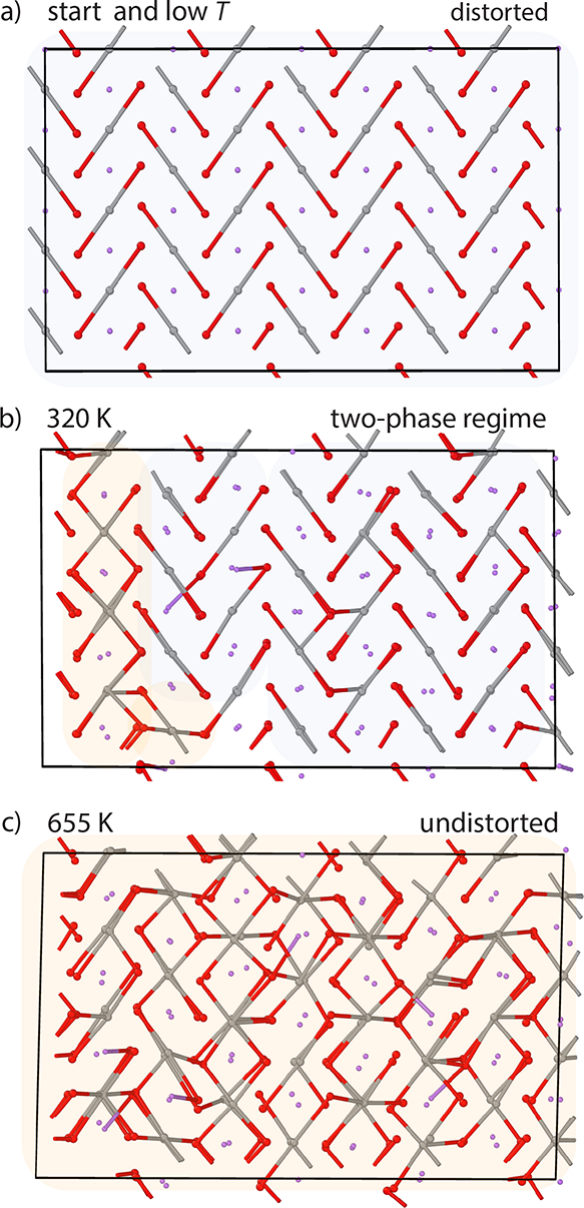
AIMD trajectories starting from a zigzag (0 K ground state) distorted structure at varying temperatures. (a) At low temperatures (*T* < 250 K), the cell stays distorted and maintains the original cooperativity despite thermal vibrations. (b) At intermediate temperatures (250 K < *T* < 350 K), undistorted domains (orange) form and coexist with distorted domains (blue). (c) At high temperatures (*T* > 350 K), the NiO_6_ octahedra within the simulation cell become fully undistorted. Ni ions are shown in gray, O ions in red, and Li ions in purple. Long Ni–O bonds (>2 Å) are drawn to aid visualization. All AIMD trajectories were run for 3 ps and are viewed along the hexagonal *c* axis.

Finite temperature effects on the structural distortions were explored with AIMD simulations starting from the ground state, zigzag cooperative distorted simulation cell at constant pressure (*p* = 0) (videos of exemplary trajectories are provided in the Supporting Information). At low temperatures (0 < *T* < 250 K), thermal vibrations are observed, but the NiO_6_ octahedra within the simulation cell stay cooperatively zigzag distorted across the simulation (Figure 2). The orientation of all distortions is maintained; i.e., no pseudorotations of the distortions are observed during the course of the simulations.

**Figure 3:**
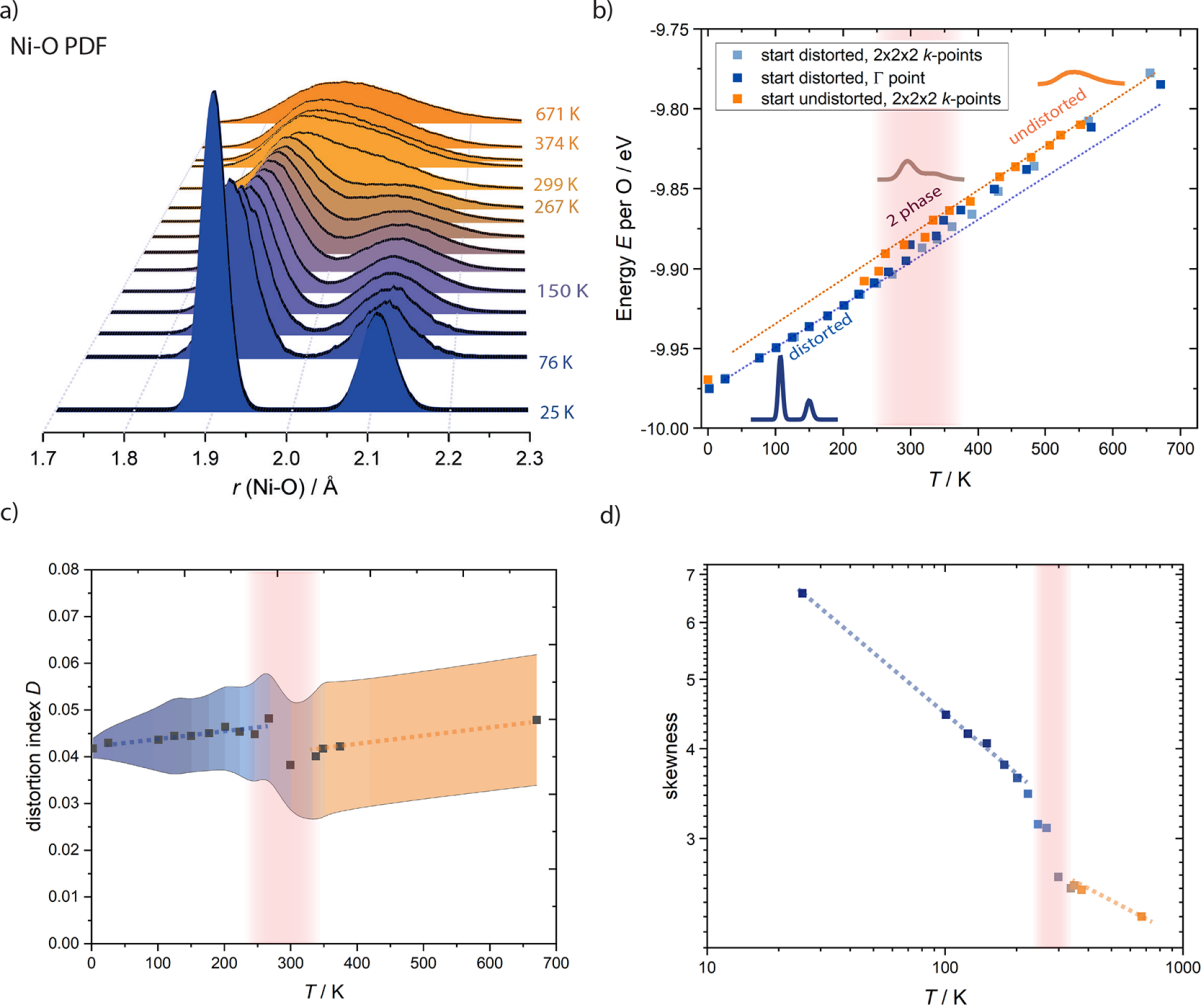
Temperature dependence of structural distortions in LiNiO_2_. (a) Simulated Ni–O PDF, (b) internal energy (*E*) of the distorted and undistorted phase as a function of temperature, (c) Ni–O bond-length distortion index (*D*) defined in eq 1, and (d) skewness of the PDF, all obtained from AIMD simulations. Blue shading denotes the distorted and orange is the undistorted phase. PDFs representative of the distorted, mixed-phase, and undistorted regimes from (a) are added to (b) to aid the phase identification. The dashed lines in (b–d) are a guide to the eye, illustrating the behavior of the pure distorted and undistorted phases. The width of the curves in (c) illustrates the range of distortions of different octahedra over time.

The Ni–O pair distribution function (PDF) provides a clear way of visualizing and quantifying the extent of Jahn–Teller distortion for all the Ni ions in the cell. The PDF in Figure 3a exhibits two peaks at low temperatures, one large peak around 1.9 Å (corresponding to the four short Ni–O bonds) and a smaller peak at 2.1 Å (corresponding to the two long bonds). The peaks become broader on increasing the temperature to ca. 250 K and begin to merge, but the PDF still continues to show clear characteristics of Jahn–Teller distortions.

From a temperature of around 250 K, changes can be seen both in the trajectories and in the PDFs. The orientation of the distortions begins to change and the distortions exhibit dynamic behavior, in line with predictions by Sicolo et al.^9^ and Middlemiss et al.^11^ The onset of the dynamic behavior is reflected in the peaks of the Ni–O PDFs being merged to an extent where the long-bond peak is discernible only as a shoulder rather than a distinct peak (Figure 3a, 267 K).

A closer look at the trajectories between 250 and ca. 350 K, however, reveals that the reorientation of the long-bond axes is not the only process of interest. At these temperatures, short-lived, undistorted domains also form that no longer exhibit two distinct sets of Ni–O bond lengths but instead a range of bond lengths. These domains form, coexist with the distorted domains (Figure 2, blue and orange domains at 320 K), and then transform back into distorted domains. While the domains are undistorted, they do not necessarily exhibit six bonds of equal length at any given time but undergo rapid changes in bond lengths, with the bond lengths varying approximately independently from each other. The lifetimes of the undistorted domains increase with temperature, ranging from a few femtoseconds at the onset of the mixed-phase regime to picoseconds around 350 K, with the domain size (correlation length) also increasing with temperature.

At temperatures above ca. 350 K, the simulation cell becomes fully undistorted, i.e., on heating the material undergoes a phase transformation. As in the case of the undistorted domains at intermediate temperatures, the fully undistorted phase exhibits dynamic behavior; the local environments change rapidly (on the femtosecond time scale), showing large variations in the bond lengths. It is no longer possible to unambiguously identify “long” bonds as the 4 + 2 metrics of the Jahn–Teller distortions are softened, yielding one continuous broad Ni–O peak in the PDF (Figure 3a, 374 and 671 K). Note, the PDF peak of the undistorted phase still exhibits a residual asymmetry signifying an average anisotropy of the octahedral bonding environments. This is at least in part due to anisotropic elastic properties of the layered structure^8^ resulting in anisotropic thermal vibrations (isotropic thermal vibrations would only cause a symmetric broadening of the PDF). As this anisotropic high-temperature phase is not Jahn–Teller distorted, we refer to this phase as the undistorted phase: even though a snapshot of an octahedron will find a range of Ni–O bond lengths, these on average center around one set of Ni–O bond lengths rather than two sets of bond lengths as in the low-temperature Jahn–Teller distorted phase.

The internal energy of the LNO simulation cell increases with temperature (Figure 3b) due to the increased thermal vibrations, showing a linear increase at temperatures below 250 K. At high temperatures above ca. 350 K, the energy of the undistorted phase also increases linearly. In the temperature regime from 250 to 350 K, where distorted and undistorted domains coexist, the energy oscillates between the extrapolated linear curves of the phase-pure distorted and undistorted phases (highlighted in Figure 3b), due to varying amounts of time spent in distorted/undistorted environments.

To shed further light on the nature of the different phases and the phase transition, we determined the bond length distortion index *D*,^30^ a measure of the deviation of the Ni–O bond lengths *l*_*i*_ from their average value *l*_av_, often used to quantify the magnitude of Jahn–Teller distortions^8^^,^^31^^,^^32^
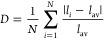
1

The bond length distortion index of the distorted low-temperature phase shows a slight increase with temperature from ca. 0.04 around 0 K to 0.045 at 250 K (Figure 3c). The increase is presumably due to thermal effects, particularly anisotropic thermal expansion, with the interlayer expansion in *c* direction expected to be the largest.^8^ This results in a subtle lengthening of the long Ni–O bond relative to the short bonds (see Supporting Information), with the short bond lengths being approximately constant across the temperature window of the distorted phase. A clear drop in distortion index to 0.035 is seen in the mixed-phase temperature regime 265 K < *T* < 350 K, where undistorted domains begin to form. Temperature and anisotropic thermal expansion then cause the distortion index in the undistorted phase to increase again. The local distortion index hence shows a clear phase transition, and the transition regime agrees very well with the mixed-phase regime identified from the energies and PDFs (Figure 3a,b).

Somewhat unexpected is the high distortion index of the undistorted phase at high temperatures. In a hypothetical scenario where every Ni ion shares six Ni–O bonds of equal length and all octahedra are the same size, the distortion index would be zero. Given thermal effects and the slight asymmetry of the Ni–O PDF (Figure 3a), a finite distortion index is expected. It is surprising, however, that at high temperatures it is comparable to the distortion index of the distorted phase, yet the trajectories (Figure 2) and PDFs (Figure 3a) show clear differences between the phases. It can be concluded that the distortion index can be a helpful means of characterizing distortions and phase transitions but needs to be complemented with other analyses.

The asymmetry of the PDF can be quantified further by determining the skewness of the distribution function, specifically the Fisher–Pearson coefficient of skewness *G*_1_(33)
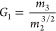
2

with moments *m*_*i*_
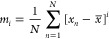
3

The skewness of the PDF (Figure 3d) monotonically decreases with temperature; i.e., the PDF becomes less asymmetric with temperature. This is also true within the undistorted regime, where higher temperature gives rise to a more symmetric PDF. As a result, the skewness in the undistorted phase at all temperatures clearly differs from the skewness in the distorted phase. A jump in the skewness is seen around 250–350 K, i.e., the skewness decreases rapidly in the mixed-phase regime around the phase transition.

The skewness and local distortion index hence consistently identify the onset of the phase transition to be at ca. 250 K, with the mixed-phase regime extending to ca. 350 K.

As the symmetry and size of the starting configuration could, in principle, impose artificial constraints on the simulations, all simulations were performed for both a distorted starting structure (8 × 2 × 2 unit cells, 256 ions) and an undistorted cell (4 × 2 × 2 unit cells, 192 ions). Both simulation cells became distorted at low temperatures (*T* < 265 K), undistorted at high temperatures (*T* > 350 K), and showed coexistent distorted and undistorted domains in the two-phase regime (Figure 3b). The energy per O in the simulation cells was nearly identical, as were the PDFs. For the distorted structure, simulations based solely on the Γ point yielded very similar energies and PDFs to those obtained with a 2 × 2 × 2 *k*-point grid but allowed for longer simulation runs (*∼*3000 fs vs ∼500 fs) resulting in better equilibration. The longer runtimes mostly improved the equilibration in the mixed-phase regime, where shorter runs (irrespective of the number of *k*-points) tend to exhibit more distorted character (as the starting structure was distorted). In the interest of statistics, the Γ-point calculations were therefore considered for further analyses. Note, for the *R*3̅*m* starting configuration, Γ-point simulations differ from higher *k*-point calculations at all temperatures.

**Figure 4:**
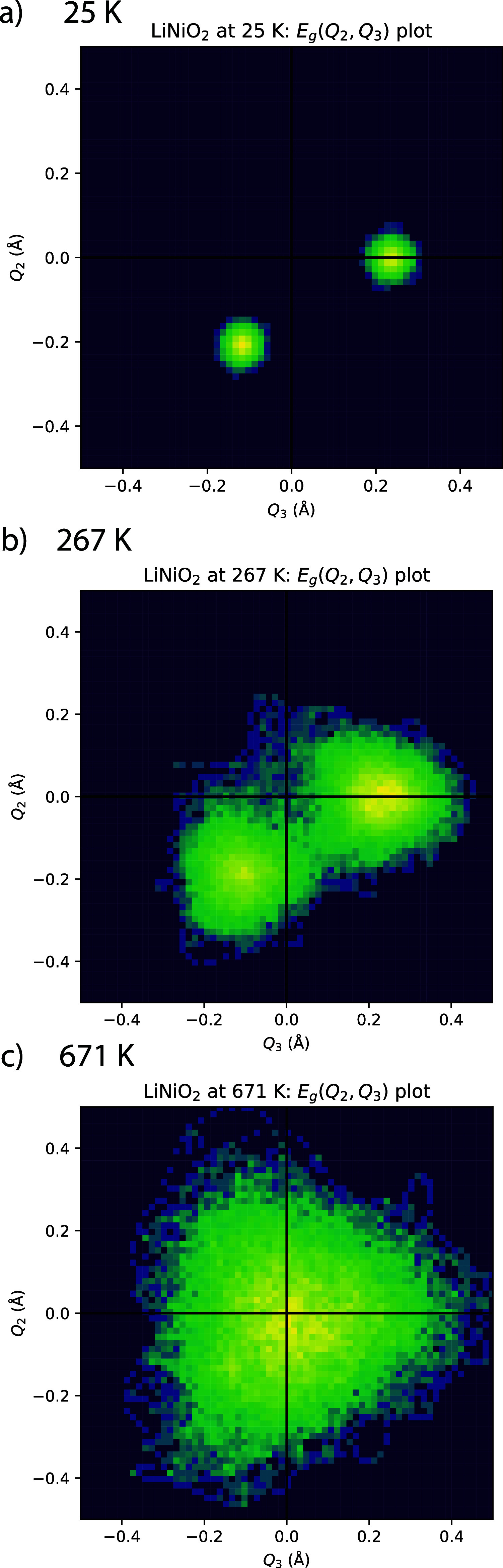
Heat map of a van Vleck analysis of the octahedral distortion modes of AIMD trajectories. Each data point represents a NiO_6_ octahedron. The magnitude of the JT distortions ρ_0_ (distance from the pole) is shown as a function of the angle ϕ which characterizes the type of distortion (0, 120, and 240° correspond to three possible orientations of the long axes of quadratically elongated octahedra in LiNiO_2_). Brighter colors denote higher probability of a distortion. (a) At 25 K, the octahedra are statically aligned along two axes (zigzag). (b) At 267 K, the orientation of the octahedra can change between the two axes. (c) At 671 K, the octahedra are not oriented along any axes, exhibit dynamic behavior, and spend most time in undistorted states (highest intensity at the pole).

A van Vleck analysis^22^ of the octahedral distortion modes yields irreducible representations of all possible distortions. This provides a graphical representation of the extent and nature of the distortions of the octahedra, being specifically sensitive to distortions with the symmetry of the *e*_g_ orbitals. In contrast to the bond length distortion index, *D*, a van Vleck analysis can therefore separate Jahn–Teller distortions from thermal anisotropies. JT distortions in LiNiO_2_ are characterized mainly by their *Q*_2_ and *Q*_3_ modes (Figure 4), with the distance from the pole ρ_0_ denoting the magnitude of the distortions and the angle ϕ the nature of the distortions. For JT distortions in LiNiO_2_, ϕ assumes values of 0, 120, and 240° corresponding to the three possible directions of the JT long axes. A van Vleck analysis of the AIMD trajectories at 25 K (Figure 4a) exhibits two isolated clusters of data points along two axes. This shows that the NiO_6_ octahedra all have distortions of similar magnitude, but the JT long axes are oriented in different directions, as found in the zigzgag structure (statically distorted octahedra in zigzag arrangement). At 267 K (Figure 4b), the data points still show the greatest density along two axes but exhibit a greater scatter due to thermal fluctuations and overlap due to transitions between the octahedra.

At 671 K (Figure 4c), the octahedra are no longer aligned to any of the three axes of the van Vleck plot that correspond to Jahn–Teller distortions but show highly dynamic behavior, with the greatest probability of finding octahedra at the pole with *Q*_2_ = *Q*_3_ = 0, i.e., in an undistorted configuration. The question arises how the transition from static to dynamic distortions occurs, and ultimately, how this relates to the dynamic undistorted high temperature phase.

### Pseudorotation Mechanism

**Figure 5:**
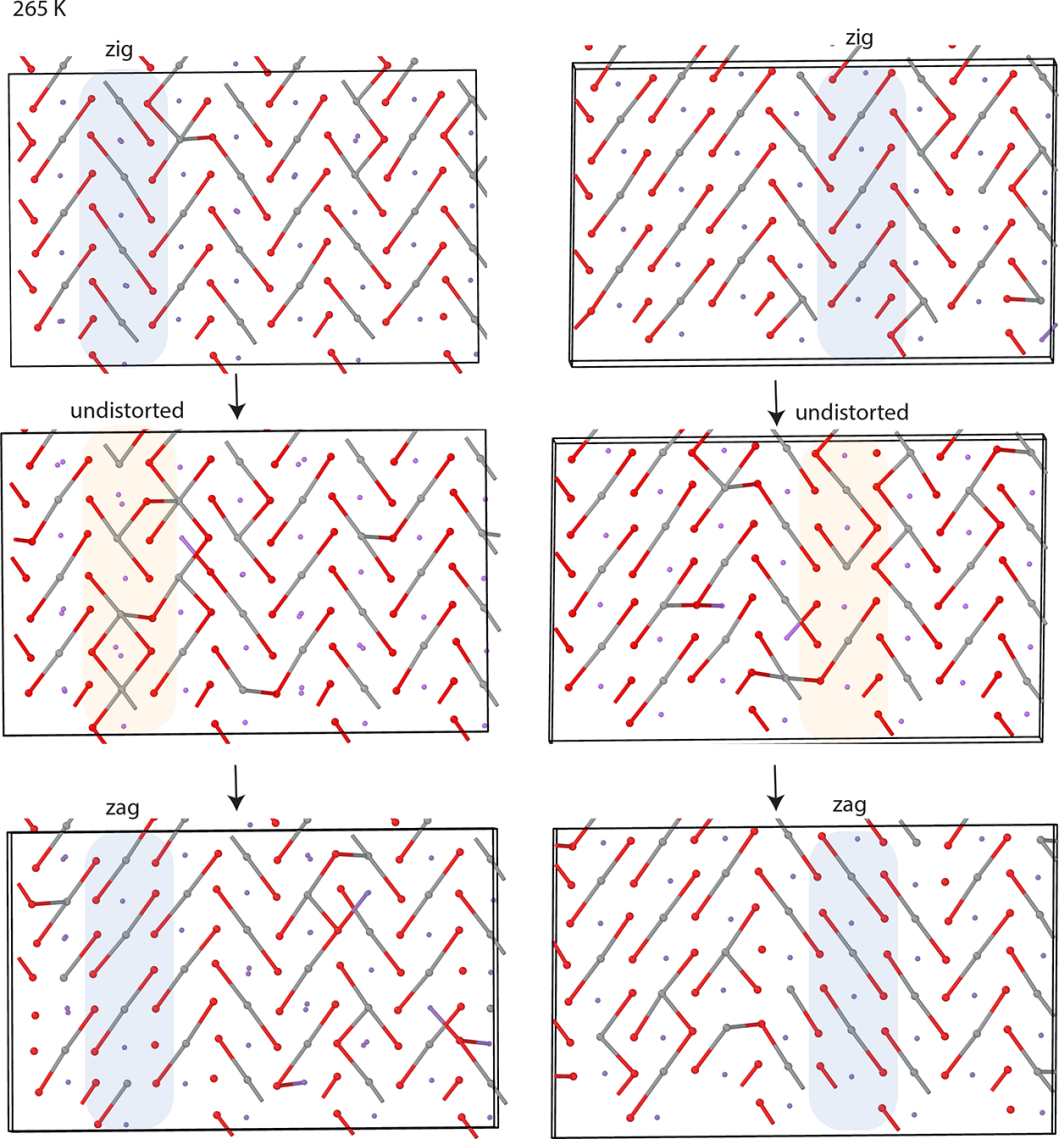
Illustration of the pseudorotation mechanism. When undistorted domains (orange) reform distorted domains (blue), the long Ni–O bonds can change direction or resume the prior direction, as shown for two pseudorotations occurring in the same trajectory. The undistorted phase does not exhibit a specific single configuration but a broad and dynamic variation in bond lengths.

To shed light on the onset of the dynamic behavior of the JT distortions (at ca. 250 K), AIMD trajectories were analyzed with respect to the mechanism of pseudorotations. Pseudorotations have been proposed by Radin et al.^1^ and Sicolo et al.^9^ to explain the dynamic behavior of JT axes. In our AIMD trajectories, this pseudorotation process was found to occur not for individual octahedra but for complete rows of equally oriented distortions (e.g., a complete zig row changes to zag) (Figure 5).

A closer look at the trajectories reveals that undistorted domains are at the heart of the reorientation process. The temperature window where dynamic Jahn–Teller distortions are observed (250–350 K) is identical with the mixed-phase regime where undistorted domains form in the distorted material and frequently revert back to a distorted state. When the distorted domains reform, the direction of the long-bond axis can either change (Figure 5) or go back to the direction of the distortions before the undistorted domain was formed. Both processes are observed in the simulations.

**Figure 6:**
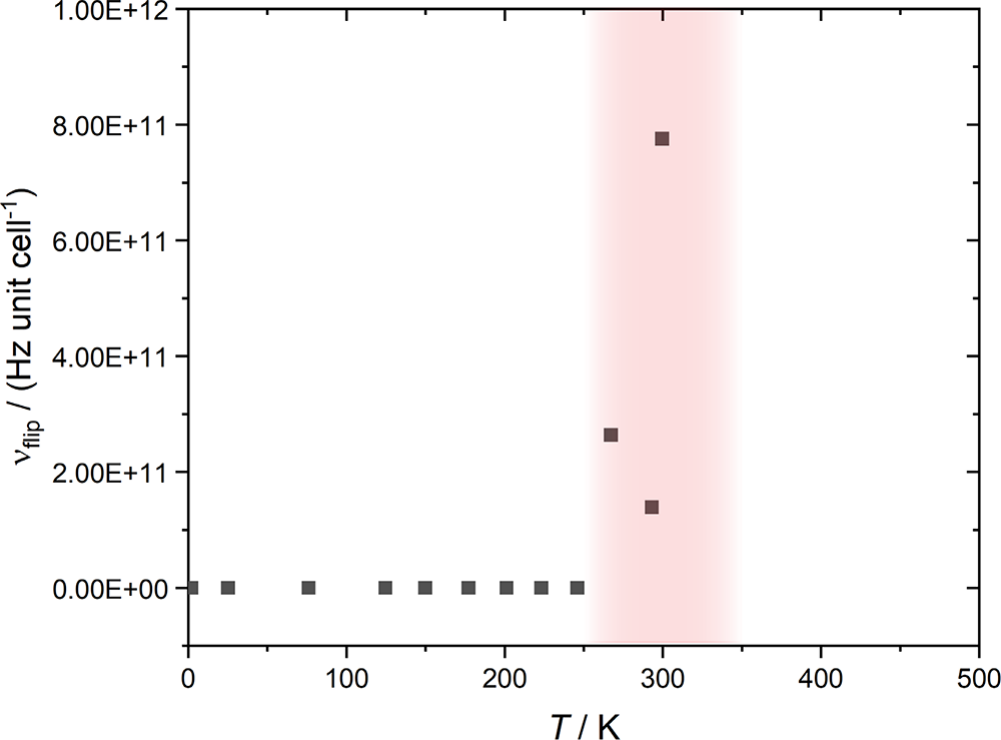
Pseudorotation frequencies, ν_flip_, as a function of temperature. The frequency (Hz per unit cell) is zero for *T* < 250 K. At *T* > 350 K, the rotation is not well-defined.

The dependence of the pseudorotations on the coexistence of distorted and undistorted domains is also reflected in the pseudorotation frequency, ν_flip_, obtained from counting the number of reorientations occurring per time in the AIMD trajectories (Figure 6). These are zero at low temperatures (*T* < 250 K) and rapidly increase in the mixed-phase regime to ca. 8 × 10^11^ Hz per unit cell. At high temperatures (*T* > 300 K), the axes of the octahedra are no longer well-defined as the six bonds vary individually in length. Finite flipping frequencies are thus only found in the mixed-phase regime.

### Antisite Defects

**Figure 7:**
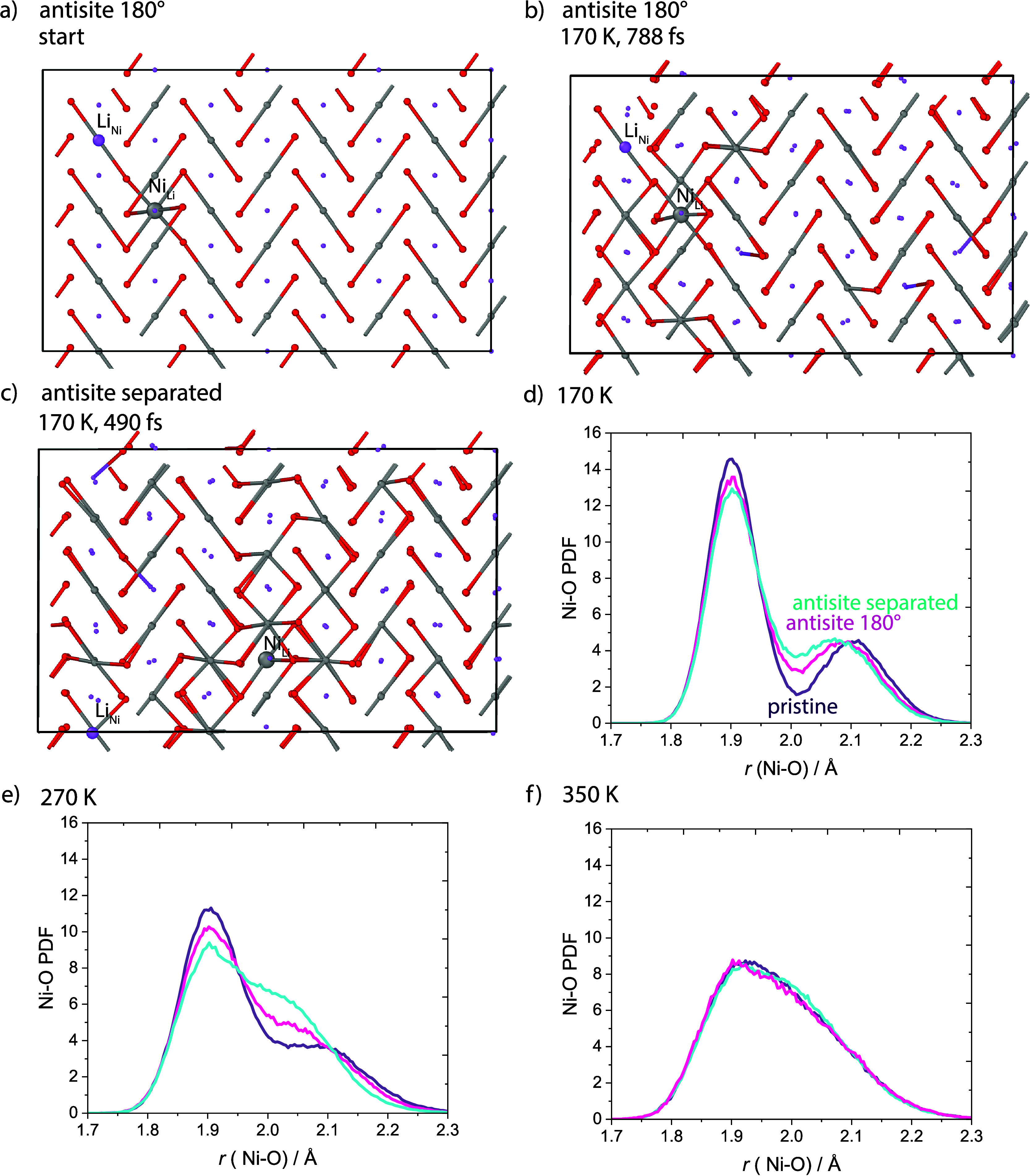
Impact of antisite defects on the Jahn–Teller distortions. (a) Starting configuration of a simulation cell with an antisite defect pair in 180° configuration, (b) snapshots of the same cell at 170 K, (c) of a cell with separated antisite defects, and (d–f) resulting Ni–O PDFs.

Antisite defects are unavoidable in LiNiO_2_ synthesized via a conventional solid-state synthesis route, and it is very likely that they affect/interact with the structural distortions in any “real” or experimental sample of LNO.^3^ To shed light on the interplay of the antisite defects and the distortions, antisite defect pairs were introduced into the simulation cells (corresponding to 2 at. % of the Li and Ni ions exchanging sites). Two different configurations were explored, one where the defects are next-nearest neighbors sharing a 180° Ni–O–Li bond (Figure 7a,b) and one where the defects are separated (Figure 7c) by 7.6 Å. We have recently proposed that the defect pair bound in a 180° configuration represents the lower energy state, but separated defect pairs could easily form at high temperatures (e.g., at synthesis conditions).^34^ As the material is delithiated, the defects could change configuration (e.g., the ions become mobile and separated defects could, for example, form pairs, or move to their proper lattice sites, *etc.*). The concentration of the respective defect pairs will, therefore, depend strongly on the thermal and electrochemical history of the sample.

At low temperatures (*T* < 250 K), the introduction of either type of defect pair creates locally undistorted environments, not only around the Ni ion in the Li layer itself but also around neighboring Ni ions and around the Li ion in the Ni layer and neighboring Ni ions (Figure 7b,c). The defects hence create undistorted domains within the distorted low-temperature phase. The resulting Ni–O PDF (Figure 7d) exhibits less intensity at the distances corresponding to the long (∼2.1 Å) and short (∼1.9 Å) Ni–O bonds in the distorted structure and significantly more intensity at the distance corresponding to the Ni–O bond length in the undistorted structure (∼2.0 Å). The PDF hence mirrors an overall less distorted character in comparison to the PDF of the defect-free material.

The same behavior is observed in the mixed-phase regime (250 K < *T* < 350 K) (Figure 7e). Comparing the impact of the different defect pairs (Figure 7d,e), the separated defects were found to cause more undistorted character of the PDFs than the defects in the 180° configuration. This is presumably due to the separated defects creating more undistorted domains (one each) as compared to the defect pair in the 180° configuration. The separated defects can therefore disrupt the distortions to a greater extent. We leave for a future study to explore further how the domain size and distribution affects the PDFs. In the high-temperature undistorted phase, the antisite defects do not affect the PDF (Figure 7f), as the pristine material is undistorted at this temperature easily accommodating the defects in undistorted environments (the PDF exhibiting one continuous slightly asymmetric peak, as discussed in the “Temperature-Dependent Phases” section).

### Ionic Charges and Spin-Magnetic Moments

Jahn–Teller distortions in LNO are typically discussed based on the assumption of a *d*^7^ electron configuration^10^ but this is not a mandatory assumption. We recently proposed that the strong covalency of the Ni–O bond results in a Ni electronic configuration close to *d*^8^ and electron holes on the oxygen. The resulting ligand-field splitting energy level scheme deviates from the simple crystal field model^2^ but also shows signs of Jahn–Teller distortions, i.e., partially occupied degenerate states resulting in a structural distortion. Thus, the overall number of electrons shared between Ni and O is not affected by how the charge density is assigned to either ion species.

**Figure 8:**
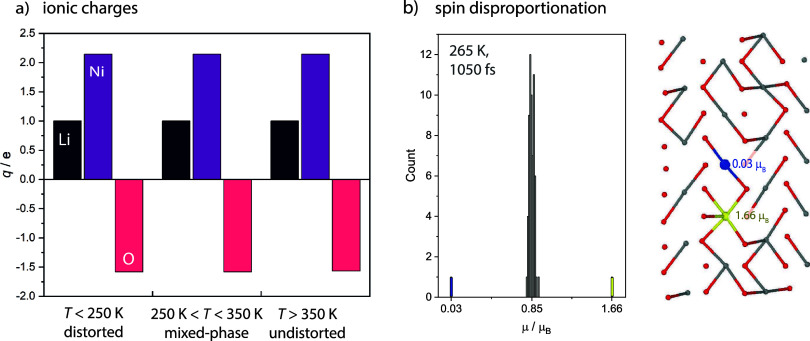
Charges and spins. (a) Ionic charges derived from the AIMD trajectories of the different phases are unaffected by the transition from the distorted low-temperature phase to the undistorted high-temperature phase. (b) Histogram of the spin magnetic moments and structure of an AIMD snapshot at 265 K showing spin disproportionation from ca. 0.85 μ_B_ (gray) to 1.66 μ_B_ (yellow) and 0 μ_B_ (blue), particularly in the undistorted domains/phase.

A recent prediction of dynamic spin disproportionation in LiNiO_2_^35^ (a dynamic model related to the spin disproportionation reported by Foyevtsova et al.^29^) suggests that the regular spin corresponding to one unpaired electron per Ni (*S* = 1/2 with a spin magnetic moment in the field direction of 0.85 μ_B_) dynamically disproportionates, resulting in some Ni ions temporarily exhibiting a spin corresponding to two unpaired electrons (1.66 μ_B_ – *S* = 1, formally Ni^2+^) and some exhibiting zero moments (*S* = 0, formally Ni^4+^), before the nickel ions with *S* = 1 and *S* = 0 comproportionate again. The question arises if such dynamic behavior of the spins could be related to the structural distortions and their respective dynamics. Similar discussions revolve around the interplay of charge/spin fluctuations and structural distortions in AgNiO_2_,^36^^,^^37^ which, crystallizing in the delafossite structure, has similar NiO_2_ layers as LiNiO_2_. However, we observe dynamic spin disproportionation in all phases and do not see evidence of an onset of the spin dynamics in relation to any of the phase transitions. When both phases are present (in the mixed-phase regime), we see a preference for disproportionation (and comproportionation) to occur in undistorted environments (Figure 8b). Ni ions with several long Ni–O bonds tend to carry the double moment, whereas the moment of a nearby Ni ion within the same row is quenched. The spin phenomena do not affect the ionic charge states of the phases, with the ionic charges of the distorted phase, the mixed phase, and the undistorted phase all corresponding to the charge states recently reported^2^ (and these are not affected by the spin disproportionation, either). This follows the trends seen previously for the charge and spin states of LiNiO_2_ on Li removal, with LiNiO_2_ and NiO_2_ both exhibiting nearly the same Ni ionic charge but different spin states due to changes in the Ni–O covalency and oxygen holes.^2^

The disproportionation and comproportionation frequencies are higher in the high-temperature phase, but it is unclear at this stage whether the frequency increases due to the disproportionation being enhanced in the undistorted structure or whether the spin dynamics are simply temperature-activated, irrespective of the structure. Note, DFT calculations are still mostly based on the electronic ground state (with some electron smearing used to imitate finite temperature effects, by allowing fractional occupancies near the Fermi level), i.e., temperature effects of the electrons are not fully captured (only those of the nuclei are fully included). We leave this for future study alongside a more in-depth investigation of the possibility of a causal relationship between the spin dynamics and the formation of the undistorted phase (answering the question of whether the undistorted phase forms because of the spin dynamics or whether the phenomena are unrelated).

### Variable Temperature (VT) XRD Studies of LiNiO_2_

**Figure 9:**
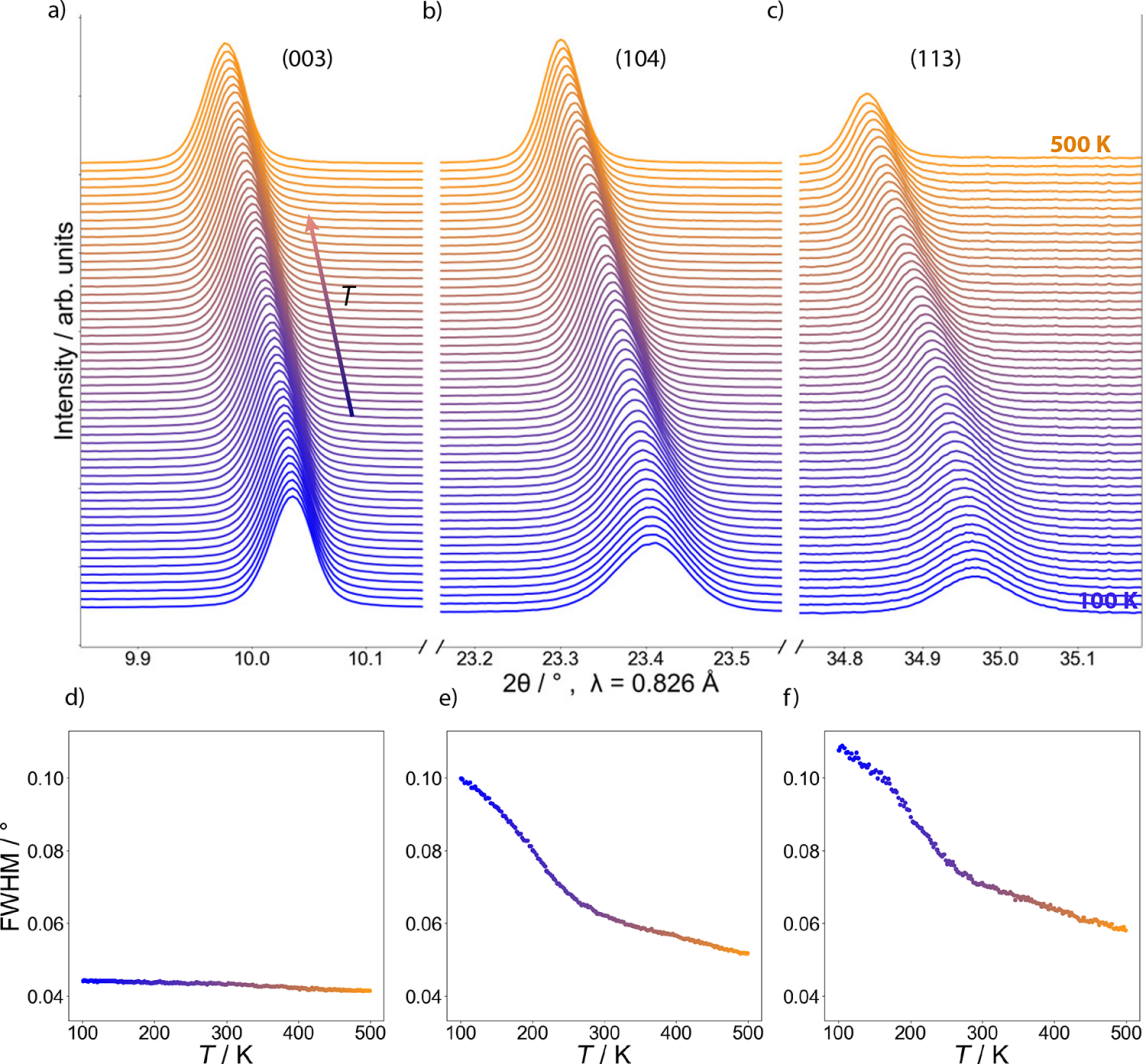
XRD peak sharpening on heating of (a) the (003) reflection, (b) the (104) reflection, and (c) the (113) reflection. The color gradient from blue to orange (and the direction of the blue-orange arrow) denotes increasing temperature from 100 to 500 K in steps of 7.5 K. (d–f) Full widths at half-maximum as a function of temperature, decreasing with temperature and exhibiting a change of slope around 250–300 K. The decrease is particularly pronounced for the (104) and (113) reflections.

VT X-ray diffractograms of LiNiO_2_ with ca. 3.8(6)% Li on Ni sites and 3(1)% Ni on Li sites were recorded at 100 K < *T* < 500 K. Interestingly, the diffractograms exhibit a sharpening of some of the peaks on heating and a broadening on cooling (Figure 9a–c, broadening on cooling in the Supporting Information). For example, as shown in Figure 9, the (104) and (113) peaks of the *R*3̅*m* structure broaden considerably, with the full width at half-maximum (fwhm) increasing by 92 and 85%, respectively, in the temperature range 100–500 K. On the other hand, the (003) peak shows only a minor change (6%). Notably, the unusual peak shape effects are most pronounced at low temperature, with a distinct change in slope of the temperature dependence of the fwhm around 250 K (Figure 9d–f).

**Figure 10:**
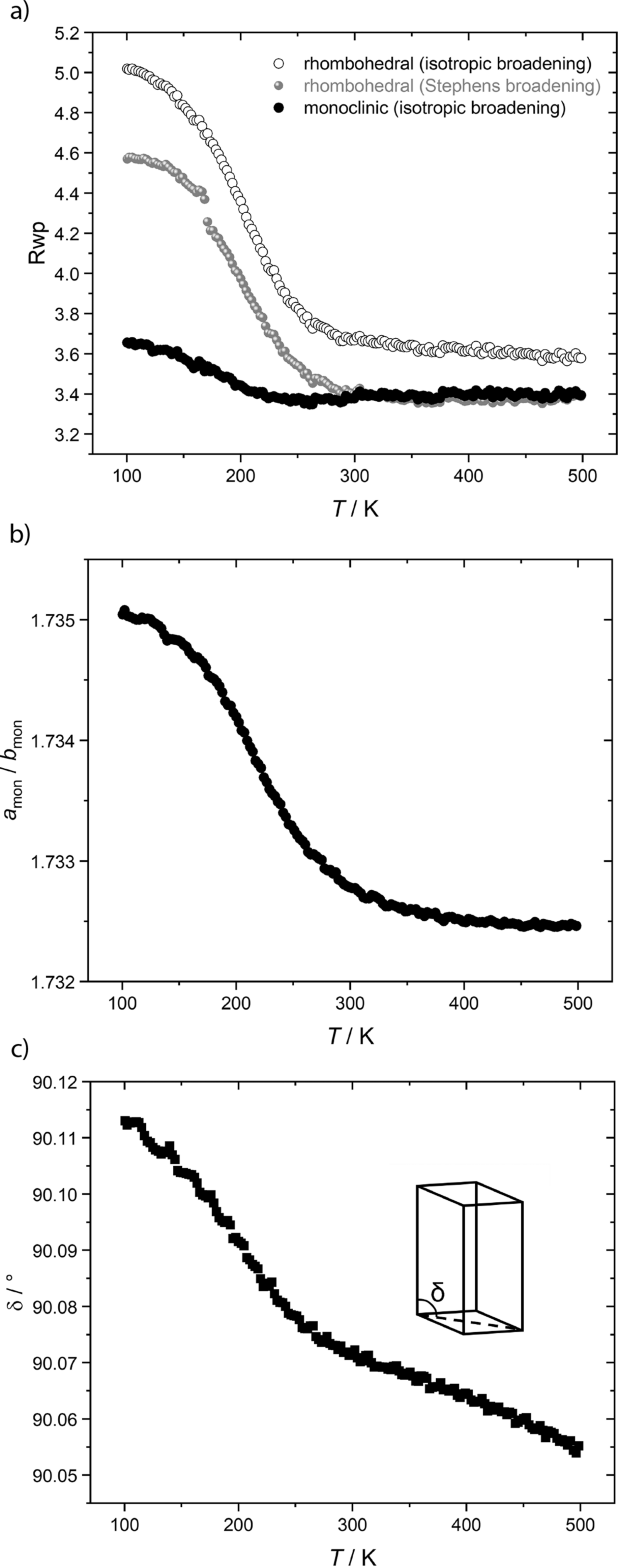
VT-XRD data in support of a structural transition around 250–300 K. (a) The *R*_wp_ value denoting the quality of the refinements based on a rhombohedral unit cell (empty symbols) with and without Stephens-type peak broadening (gray) and with the monoclinic unit cell (filled symbols). (b) Ratio of the monoclinic lattice parameters *a*_mon_/*b*_mon_ as a function of temperature, showing a drastic decrease at around 300 K. The errors are smaller than the symbol size. (c) δ angle (sketched in the inset) as a function of temperature, exhibiting a change in slope around 250–300 K.

Often, anisotropic peak broadening arises from anisotropic strain effects on the crystallites. If this were the cause of the broadening seen here, the peak widths must follow a *hkl*-dependence consistent with the symmetry of the rhombohedral structure, as described by Stephens.^38^ However, fits using the Stephens peak shapes for a rhombohedral structure fail to describe the *hkl*-dependence of the peak widths (see Figure 10a), showing little improvement compared to a rhombohedral fit with isotropic broadening. Instead, the broadened peaks were found to correspond only to the rhombohedral peaks that would be split by a symmetry lowering to monoclinic *P*2_1_/*c*, i.e., the 10*l* and 11*l* peaks. Indeed, we find that the pattern fits (measured by *R*_wp_, Figure 10a) can be greatly improved by modeling the patterns with the zigzag *P*2_1_/*c* structure (even with isotropic peak broadening). Some improvement may be expected due to the increased number of degrees of freedom of the lower symmetry model. At temperatures above 300 K, the improvement is relatively minor but rapidly becomes pronounced at temperatures below 250 K.

To track the monoclinic character as a function of temperature, we identified the symmetry-allowed distortion modes of the monoclinic structure relative to the parent rhombohedral structure (see Supporting Information). We found that four distortion modes describe the degrees of freedom of the monoclinic structure, of which two modes break the rhombohedral symmetry (the other two correspond to an interlayer and intralayer expansion of the rhombohedral cell by varying the *c* and *a* lattice parameters, respectively). One symmetry-breaking mode changes the ratio of the monoclinic lattice parameters *a*_mon_/*b*_mon_^39^ (constrained to equal  = 1.732 in the rhombohedral cell; Figures 1a and 10b) and breaks the trigonal rotational symmetry of the *R*3̅m unit cell by varying the Ni–Ni–Ni γ angle from 120°. The other mode changes the δ angle^6^ (Figure 10c) corresponding to a monoclinic shear. Previous studies have only reported changes of the δ angle^6^ but our analysis shows that *a*_mon_/*b*_mon_ and the δ angle together fully capture the transition from the rhombohedral to the monoclinic structure. We therefore extracted the *a*_mon_/*b*_mon_ ratio and the δ angle as a function of temperature from the *P*2_1_/*c*-based Rietveld refinement (Figure 10b,c). We find that both quantities decrease abruptly above 250 K, demonstrating a loss of the monoclinic character at those temperatures where a rhombohedral refinement also captures the structure well (Figure 10a).

To confirm these findings, complementary refinements of the diffractograms using the distortion modes as an alternative basis set were performed (see Supporting Information). The degree of the two symmetry-breaking distortion modes decreases abruptly around 300 K and approaches zero at high temperatures, confirming the loss of monoclinic distortions.

This analysis hence shows that the diffraction data above 300 K can be reasonably well described by an undistorted rhombohedral model. As LNO is cooled below 250–300 K, the rhombohedral cell no longer can describe the pattern and distortions consistent with a monoclinic structure are seen. Notably, the observed monoclinic distortions are small, and the peaks which broaden are never observed to distinctly split in the temperature range observed (unlike in NaNiO_2_, where the observed monoclinic structure shows a larger monoclinic distortion and the peaks are observed to clearly split). This likely indicates that the monoclinic distortions seen in LNO are of short coherence length; i.e. they are only present in small domains.

## Discussion

All AIMD and VT-XRD data consistently show a phase transition from a statically distorted JT-active low-temperature phase (*T* < 250 K), via a mixed-phase regime (250 K < *T* < 350 K) with coexistent distorted and undistorted domains, to a dynamic high-temperature phase (*T* > 350 K) with no long-range distortions. The undistorted phase is dynamically stabilized. There are still short-range distortions as seen by the asymmetry of the PDFs but these are not Jahn–Teller distortions, as is evident from the van Vleck analysis (Figure 4). Instead, the van Vleck diagram exhibits the greatest probability at the pole, which is characteristic of the absence of Jahn–Teller distortions. As a result, the oxygen positions in the average structure are better described by thermal parameters centered around the undistorted position rather than split position sites. We ascribe the remaining asymmetry of the PDF to anisotropic thermal vibrations. As the high-temperature phase has no Jahn–Teller distortions, we refer to it as “undistorted” throughout this article (see also discussion of the temperature dependence of the phases above).

The coexistence of distorted and undistorted domains and the transformation to the fully undistorted structure at high temperature agrees very well with the observation of a phase transition from a distorted (monoclinic) to an undistorted (rhombohedral) high-temperature phase in NaNiO_2_ by Nagle-Cocco et al.,^8^ Dick et al.,^40^ and Sofin and Jansen.^41^ The authors of these studies also report a mixed-phase regime around the transition in NaNiO_2_ based on the monoclinic and rhombohedral phase fractions found with VT neutron and X-ray diffraction. The phase transition occurs at higher temperatures than for LiNiO_2_, in line with the larger Jahn–Teller stabilization energy in NaNiO_2_^1,10^ and the mixed-phase regime and undistorted regime are shifted to higher temperatures (465 K < *T* < 495 K for the mixed-phase regime and *T* > 495 K for the undistorted phase). Otherwise, the phase behavior of LiNiO_2_ and NaNiO_2_ is qualitatively similar.

The phase transition in LiNiO_2_ is directly evident from the temperature dependence of the energy, as determined from the AIMD simulations. At low temperatures, the energy of the distorted phase increases linearly (Figure 3b). At high temperatures, the energy of the undistorted phase also exhibits a linear increase. In the mixed-phase regime, the energy oscillates between the curves of the pure phases, depending on how much time is spent in distorted/undistorted environments.

The phase transition is also reflected in the simulated Ni–O PDFs. The PDF shows two distinct peaks at low temperatures: one corresponding to the two long Ni–O bonds (∼2.1 Å) and the other to the four short Ni–O bonds (∼1.9 Å) of the statically distorted structure. The two peaks merge around the bond length of an undistorted Ni–O bond (∼2.0 Å) in the mixed-phase regime. At high temperatures, a single broad, slightly asymmetric peak shows the effectively undistorted character of the structure. Both the local distortion index and the asymmetry of the Ni–O PDF (the skewness) show an abrupt decrease between 250 and 350 K, confirming the temperature window of the two-phase regime.

The variable-temperature X-ray diffractograms directly support the computational predictions: the peak sharpening observed on heating is unusual but fully consistent with the loss of monoclinic distortions on heating and the growth of undistorted domains. The observed peak broadening on cooling suggests that this monoclinic domain formation is reversible. Stephens-type refinements showed that the broadening at low temperatures was not caused by anisotropic strain in the rhombohedral structure but by the formation of domains with monoclinic character. Note, domains are only detectable with XRD if their coherence length is larger than a few nanometers,^42^ whereas the AIMD simulations cover length scales up to ca. 1 nm. The domain sizes accessible to the two methods hence differ slightly, but the techniques are expected to complement each other well, with domains being discernible to a smaller length scale in the AIMD simulations. Note that while even the average structure probed with the XRD Rietveld analysis shows clear signs of a structural transition around 300 K, further support could be provided by a local structure probe, such as the X-ray and neutron PDF analysis, the latter having been performed by Chung et al.^6^ Chung et al. also report domain formation in LiNiO_2_ at low *T* based, first, on neutron diffractograms showing peak sharpening on heating^6^ and, second, on an inversion of neutron PDF peak heights at around 80–100 Å: while the short-range peaks below 50 Å decrease with temperature due to thermal vibrations, the long-range peaks beyond 100 Å increase with temperature. This unusual effect is consistent with the loss of domains on heating. Chung et al. suggest the origin of the domains lies in strain fields of frustrated, trimer-ordered Jahn–Teller distortions, which cause the NiO_2_ layers to be curved, this domain structure resulting in domains of oppositely aligned curvature. The trimer model, however, has since been shown to be energetically unfavorable,^10^ and Chung et al.^6^ noted themselves that the collinearly distorted *C*2/*m* model provided an equally good fit to the neutron PDF data, particularly for the first asymmetric peak corresponding to the Ni–O distances. The neutron data hence fully support our VT-XRD observations of the formation of domains with monoclinic character on cooling LiNiO_2_. Further support for the predicted phase transition based on our VT-XRD data is seen in the fit quality of the refinement based on an undistorted unit cell: the fit quality is poor at low *T* as the structure cannot capture the local distortions, but the fit rapidly improves around ca. 270 K (i.e., the fit parameter *R*_wp_ decreases, Figure 10a), as undistorted domains grow. Furthermore, the monoclinic lattice parameter ratio *a*_mon_/*b*_mon_ and δ angle derived from refinements based on a monoclinic unit cell show a clear change in temperature dependence around 300 K, approaching their values in the rhombohedral structure at high temperatures (Figure 10b,c). The VT-XRD data thus consistently support a rhombohedral high-temperature phase that forms domains with monoclinic character on cooling below ca. 300 K.

The distortions are predicted to be cooperative at low temperatures (below 250 K) at least on the length scale of the simulation cells and presumably also on a larger scale, as seen by the VT-XRD peak broadening on cooling. The absence of more immediate experimental signatures of cooperative distortions (such as a macroscopic change of symmetry observed for NaNiO_2_ or the absence of monoclinic distortions with larger coherence lengths both in our VT-XRD data and the neutron diffraction data reported by Chung et al.^6^), however, suggests that additional factors are at play.

Key suspects here are antisite defects. Whereas a defect-free synthesis of NaNiO_2_ is possible, attempts to synthesize defect-free LiNiO_2_ via conventional solid-state synthesis have to date proven unsuccessful. A synthesis route via a Na^+^/Li^+^ ion exchange of NaNiO_2_ was recently reported to yield samples with negligible Ni_Li_ concentrations,^43^ raising new questions, however, regarding the role of Na_Li_ defects remaining in LiNiO_2_ after the exchange. XRD refinements of our samples obtained from a solid-state synthesis yielded antisite concentrations of ca. 3.8(6) at.% Li in the Ni layer and 3(1) at.% Ni in the Li layer. Antisite defects in layered oxides are commonly believed to pin domains of different Jahn–Teller distortions.^6^ Our AIMD simulations with 2% antisite defects, however, reveal a greater tendency of the defects to pin the undistorted phase than the distorted one (Figure 7). Even though the defect-free material would have a proclivity to form cooperative distortions at low temperatures, the antisite defects pin (metastable) undistorted domains at low temperatures. These undistorted domains disrupt a more cooperative ordering of the distorted domains. The antisite defects hence effectively extend the mixed-phase regime to lower temperatures. In NaNiO_2_ in the absence of antisite defects, the cooperative ordering can proceed without disruptions. The extent to which the mixed-phase regime is extended to lower temperatures is expected to depend on a range of parameters including, for example, the concentration and distribution of the antisite defects, which, in turn, depend on the thermal and electrochemical history of a sample. It is conceivable that some planar defects such as different surface facets or (twin) grain boundaries^44^ may have a similar effect on the material as the antisite defects (point defects), locally stabilizing the undistorted structure, meriting further study. We leave it for a future study to explore how delithiation affects the structural distortions. Calculations of the fully delithiated material NiO_2_ show the absence of Jahn–Teller distortions (even at zero Kelvin),^2^ suggesting a general trend of the material becoming less Jahn–Teller distorted on delithiation. A notable exception here is the monoclinic phase forming at ca. 30–60% of delithiation,^3^ and it will be pivotal to explore the role of Jahn–Teller distortions in this loss of symmetry.

Methodologically, AIMD simulations including the postanalysis of the local distortion index and the skewness of the Ni–O PDF have proven a valuable tool for analyzing the temperature dependence of the structural distortions, from the atomistic level to the characterization of domain structures in the mixed-phase regime, and the loss of the distortions altogether. The van Vleck analysis of the AIMD trajectories is particularly helpful in identifying the nature of the distortions. The combined AIMD + VT-XRD approach used here to characterize Jahn–Teller distortions in LiNiO_2_ could provide insights into Jahn–Teller distortions in other materials, such as layered oxides (e.g., *A*MnO_2_) or perovskite oxides (e.g., LaMnO_3_), more generally.

As different types of phase transitions could give rise to different types of undistorted high-temperature phases,^1^ the question arises as to what type of phase transition and high-temperature phase are observed in LNO. Given that the high-temperature phase is highly dynamic and oscillates around the undistorted bond lengths, the high-temperature phase exhibits strong displacive character. It does not appear Jahn–Teller distorted at any given time; i.e., snapshots of the structure do not exhibit local Jahn–Teller distortions. This differs from the case of an order/disorder high-temperature phase, which would appear locally Jahn–Teller distorted in single snapshots and only over time average to an undistorted structure.

The free energy landscape is shaped by competition between the electronic (distorted) ground state and the lattice vibrations. At low temperatures, electron–phonon coupling is so strong that the ion positions align with the electronic ground state, resulting in distortions. In order–disorder high-*T* phases, the coupling is weaker but persists; even though the vibrations cause pseudorotations of the distortions, the local distortions are always recovered. In high-temperature displacive phases, however, the vibrations dominate the free energy landscape, washing out the minima of the distortions and resulting in one (albeit broad) free energy basin, with the greatest probability of finding the octahedra at the high-symmetry (undistorted) center of the basin. The phonons thus suppress cooperative Jahn–Teller distortions at high temperatures.

A displacive phase transition in LiNiO_2_, however, raises queries regarding the dynamic behavior of the distortions observed at temperatures around 265 K. Pseudorotations of the distortions are typically ascribed to an order/disorder phase transition, as by Sicolo et al. based on their AIMD simulations of LiNiO_2_^9^ and in line with Radin et al. predicting an order/disorder phase transition in LiNiO_2_.^1^ Could LiNiO_2_ be undergoing both types of phase transition, first an order/disorder transition from a statically distorted phase to a dynamically distorted phase (around 250 K), followed by a displacive transition (around 350 K) to the “undistorted” material?

We believe all computational and experimental data can be explained in terms of the displacive high-temperature phase alone without the necessity to postulate a third dynamically distorted phase. The regime where dynamic behavior of the distortions is observed coincides exactly with the mixed-phase regime, and the undistorted domains forming in the distorted material are, in our understanding, the cause of the pseudorotations. As the undistorted domains form and revert to the distorted state frequently, the distortions can either change orientation or maintain their orientation from before the undistorted domain was formed, as seen in the AIMD simulations. The mixed phase regime is the only temperature window where we obtain finite reorientation frequencies (Figure 6).

An ideal order-disorder phase would exhibit uncorrelated pseudorotations of individual octahedra. We observe more correlated reorientation processes. They consistently start around undistorted domains and result in complete rows of distortions changing orientation (e.g., all zig to all zag, Figure 5). If snapshots are taken with longer intervals between them, the behavior can easily be mistaken for rotations characteristic of the order–disorder phase. With increasing temperature, we see the undistorted domains growing in size and existing longer before they vanish to form the distorted domains again. What might appear as an undistorted transition state around 250 K hence gains more and more intermediate character before the undistorted transitions prevail and the distortions vanish altogether.

Note, if smaller simulation cells are used, all Ni ions reorient simultaneously through a configuration with four intermediate bonds, the only approximately undistorted configuration that the symmetry and enforced periodicity allow for. When the cells are constructed sufficiently large, however, a variety of different undistorted environments can induce reorientation of the distortions (Figure 5).

While the Jahn–Teller distortions reorient dynamically, the dynamic behavior cannot be linked to an order/disorder high-temperature phase but is rather a side-effect of the onset of the displacive phase transition with a mixed-phase regime. It is the dynamic formation and annihilation of undistorted domains that allows the distortions to change direction with respect to their prior direction. It is very likely that displacive phase transitions with a mixed-phase regime generally exhibit such pseudo-order/disorder characteristics, e.g., requiring displacive domains to enable the disorder, and the disorder exhibiting stronger correlations than typically expected for the disordered phase of a true order/disorder transition.

## Conclusions

Our AIMD and VT-XRD data consistently show static Jahn–Teller distortions in LiNiO_2_ at low temperatures (*T* < 250 K), a mixed-phase regime with coexistent distorted and undistorted domains at around room temperature (250 K < *T* < 350 K), and an undistorted displacive high-temperature phase (*T* > 350 K).

Dynamic behavior of the distortions is observed in the mixed-phase regime where domains frequently alternate between the distorted and undistorted state. When undistorted domains (re)form distortions, the distortions can change direction or resume their prior orientation. The pseudorotations therefore do not indicate a true order/disorder transition but are a side effect of the mixed-phase regime of the displacive phase transition, and we suggest displacive phase transitions with mixed-phase regimes could generally exhibit such pseudo-order/disorder characteristics.

We find that antisite defects pin the undistorted domains at low temperatures. The defects thus effectively extend the mixed-phase regime to lower temperatures, preventing long-range cooperative ordering, thus providing an explanation for the absence of experimental signatures of cooperative Jahn–Teller distortions in LiNiO_2_.

## Supporting Information Available

Temperature dependent structure of undistorted starting configuration, anisotropic thermal broadening, spin disproportionation, VT-XRD refinements, refinements based on symmetry-allowed distortions, peak broadening on cooling (PDF)
AIMD trajectory of LiNiO_2_ at 25 K (MP4)
AIMD trajectory of LiNiO_2_ at 267 K (MP4)
AIMD trajectory of LiNiO_2_ at 671 K (MP4)
