# Supplementary material for: Jahn–Teller Distortions and Phase Transitions in LiNiO2: Insights from Ab Initio Molecular Dynamics and Variable-Temperature X-ray Diffraction
Source: Chem Mater. 2024 Feb 20;36(5):2289–303. doi: 10.1021/acs.chemmater.3c02413 (PMC10938510; doi:10.1021/acs.chemmater.3c02413)
Supplement: Supplementary file 1 — cm3c02413_si_001.pdf [file cm3c02413_si_001.pdf]

Jahn-Teller Distortions and Phase Transitions in $LiNiO_2$: Insights from *Ab Initio* Molecular Dynamics and Variable-Temperature X-ray Diffraction

Annalena R. Genreith-Schriever,[1,4] Alexandra Alexiu,[1] George S. Phillips,[1,4] Chloe S. Coates,[1,4] Liam A. V. Nagle-Cocco,[2,4] Joshua D. Bocarsly,[1,3,4] Farheen N. Sayed,[1,4] Siân E. Dutton,[2,4] and Clare P. Grey,[1,4]*

[1]Yusuf Hamied Department of Chemistry, University of Cambridge; Cambridge CB2 1EW, UK

[2]Cavendish Laboratory, University of Cambridge; Cambridge CB3 0HE, UK

[3]Department of Chemistry, University of Houston; Houston, TX 77204-5003, USA

[4]The Faraday Institution, Harwell Science and Innovation Campus; Didcot OX11 0RA, UK

[*]Correspondence: cpg27@cam.ac.uk

Supporting Information

Temperature dependent structure of undistorted starting configuration

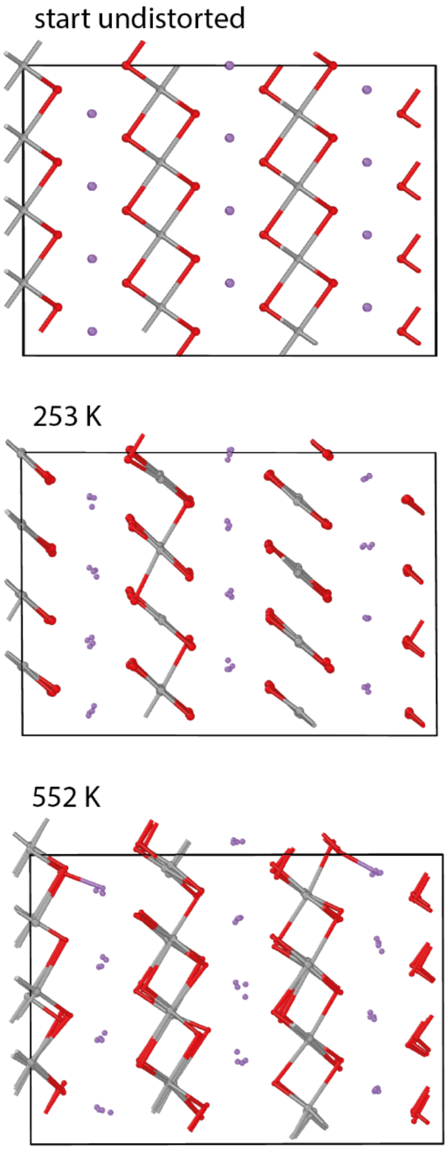

Figure S1. Impact of temperature on the undistorted starting structure. (a) An undistorted $R\bar{3}m$ starting cell turns distorted at low T (253 K) (b) and undistorted at high T (552 K) (c), comparable to a distorted starting cell.

Anisotropic thermal broadening

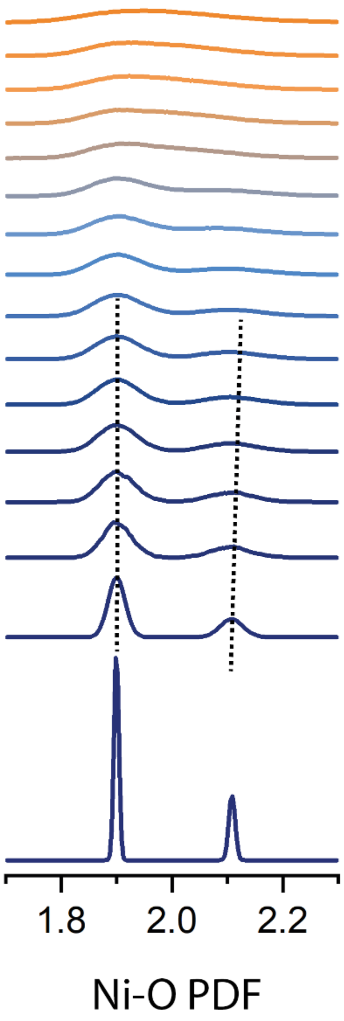

Figure S2. Impact of heating on Ni-O pair distribution function. Anisotropic thermal expansion is seen, with the long Ni-O bonds increasing more with temperature than the short bonds.

Spin disproportionation

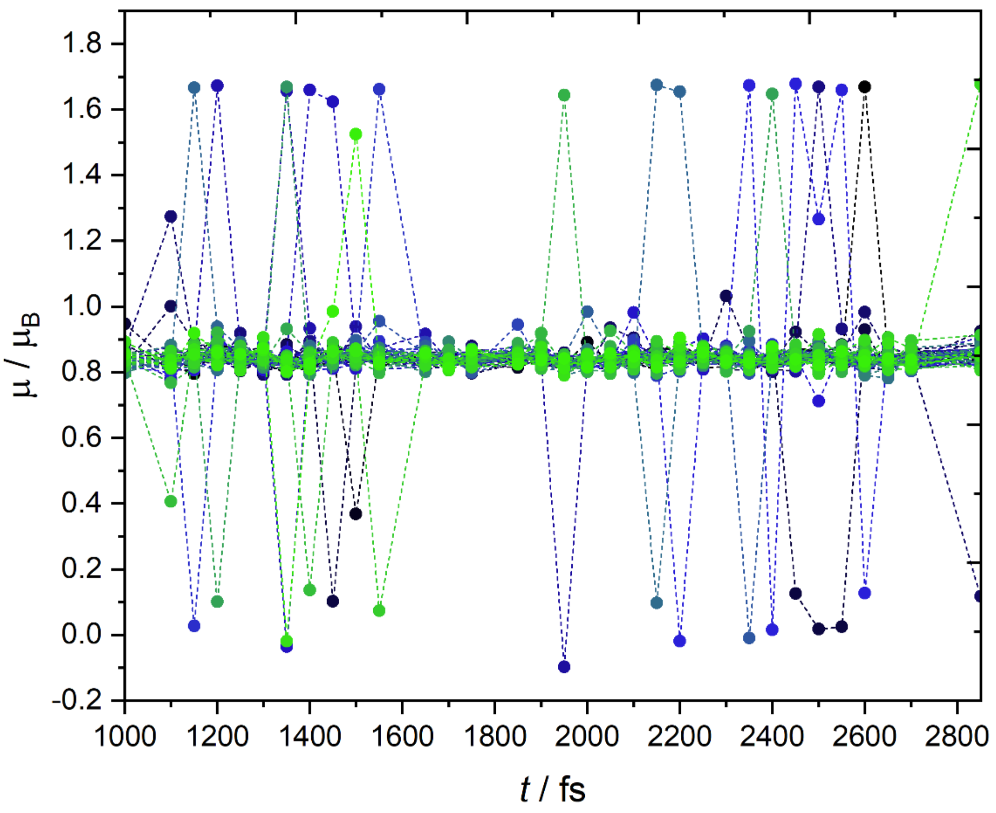

Figure S3. Magnetic moments of all Ni ions as a function of time. Spin disproportionation from a magnetic moment of 0.87 μ_B in the field direction (corresponding to 1 unpaired e^- per Ni) to 1.66 μ_B (2 unpaired e^-) and 0 is seen in AIMD simulations (with a timestep size of 1 fs and spins sampled every 50 fs).

VT-XRD refinements

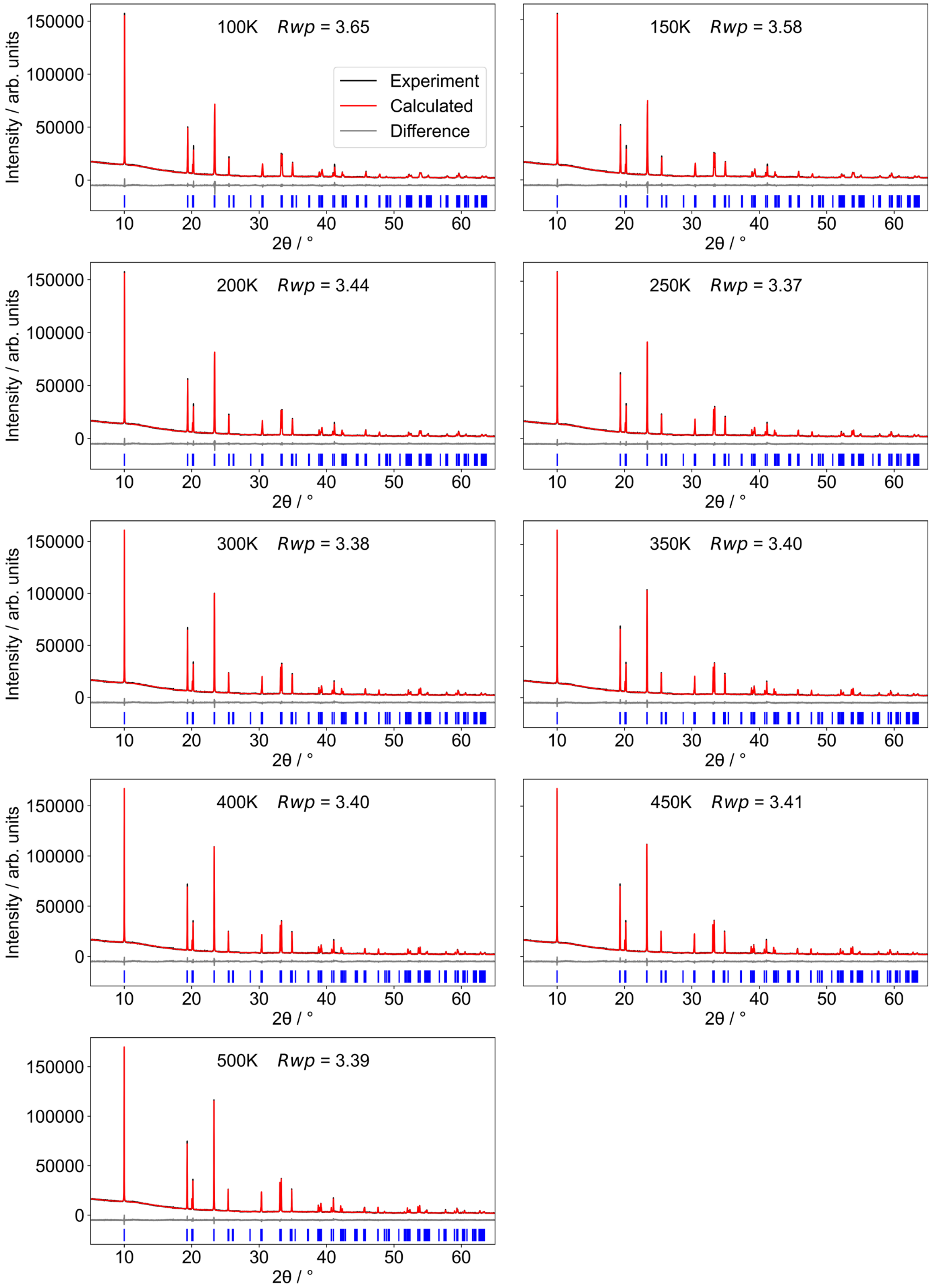

Figure S4. VT-XRD refinements based on a zigzag distorted monoclinic structure.

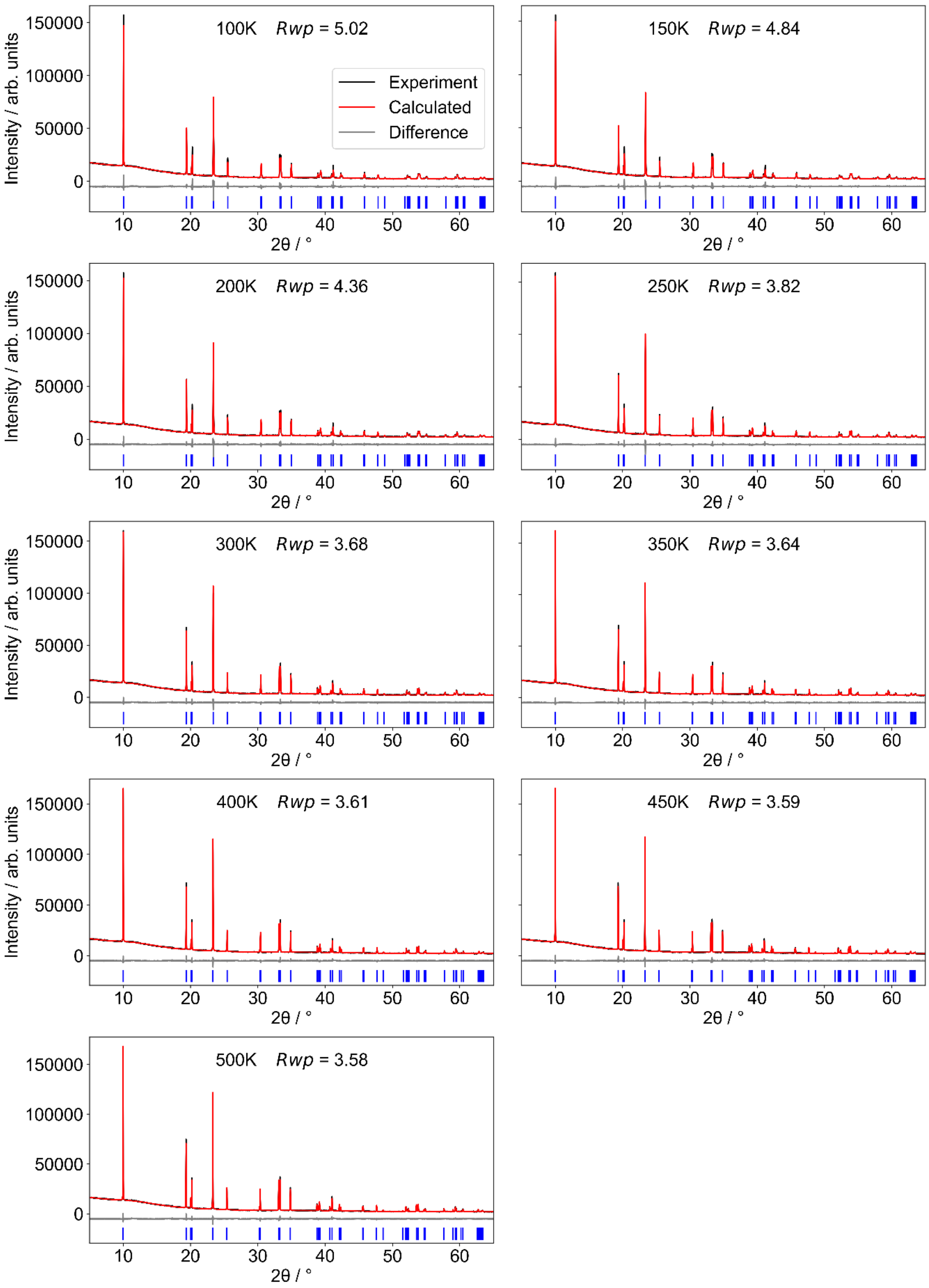

Figure S5. VT-XRD refinements based on an undistorted rhombohedral structure.

(003)

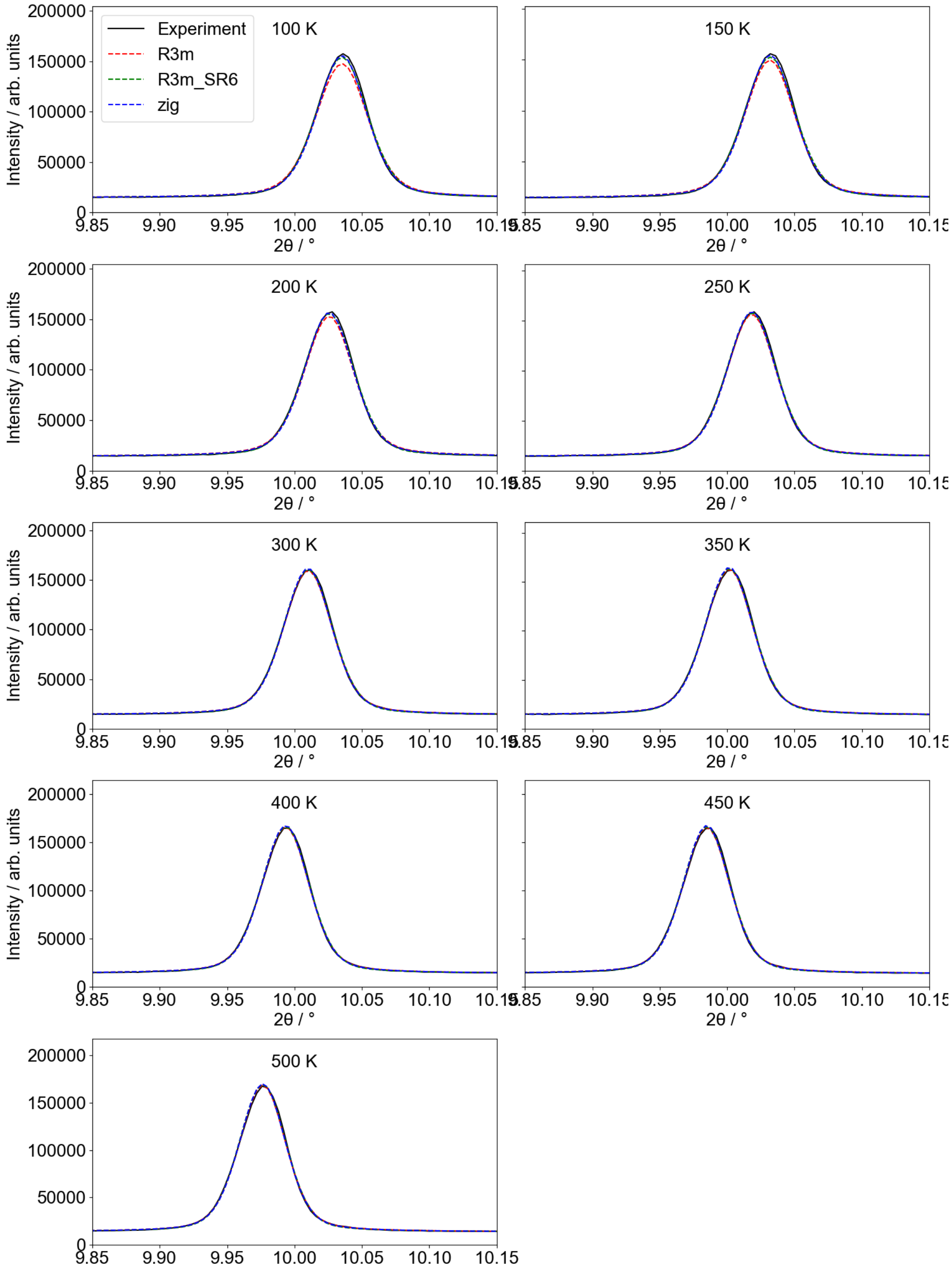

Figure S6. Comparison of the experimental (003) reflection (solid black line) with refinements based on a rhombohedral unit cell with isotropic broadening (red dashed line), rhombohedral unit cell with Stephens broadening (green dashed line), and monoclinic unit cell (blue dashed line).

(104)

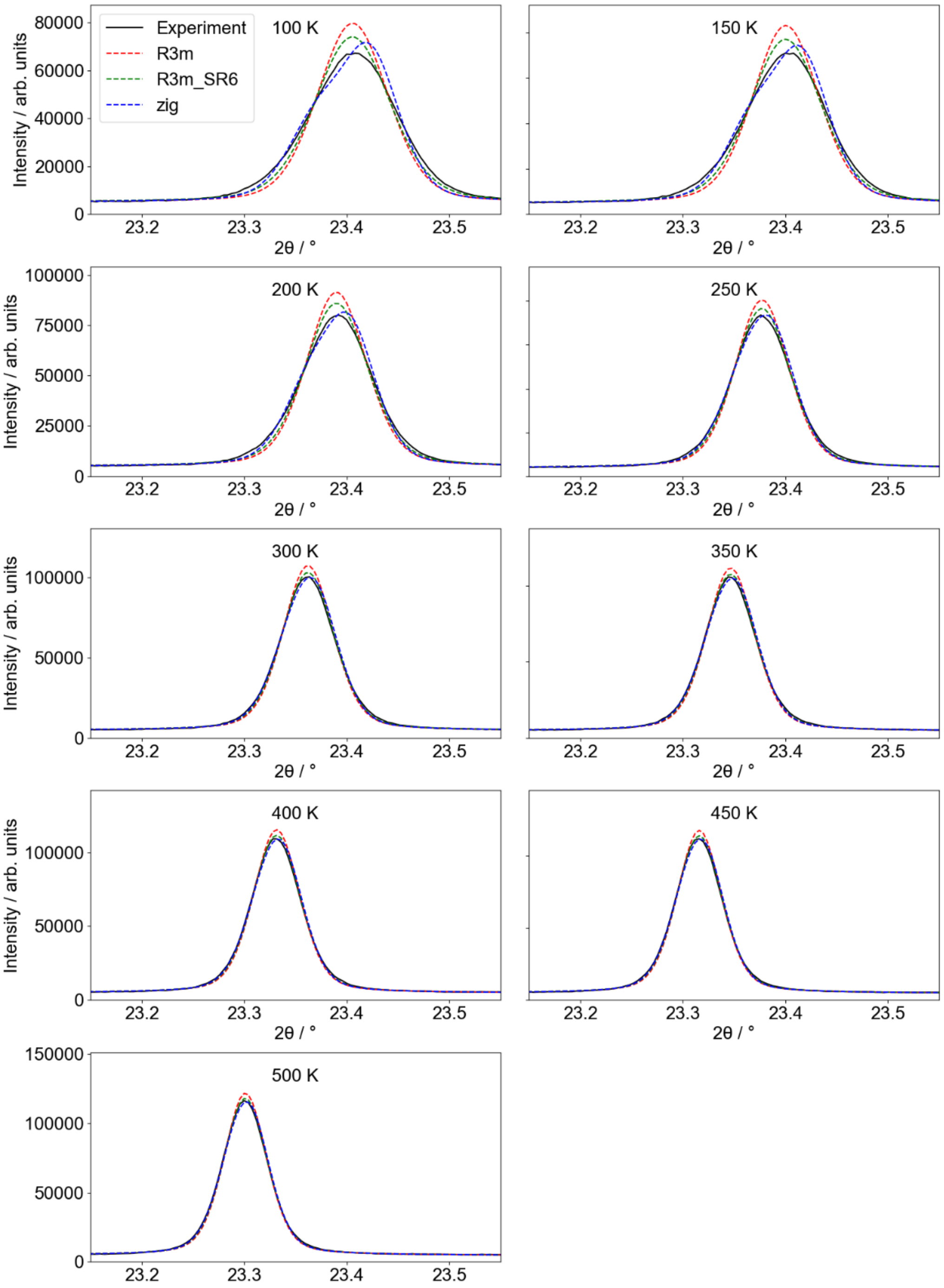

Figure S7. Comparison of the experimental (104) reflection (solid black line) with refinements based on a rhombohedral unit cell with isotropic broadening (red dashed line), rhombohedral unit cell with Stephens broadening (green dashed line), and monoclinic unit cell (blue dashed line).

(113)

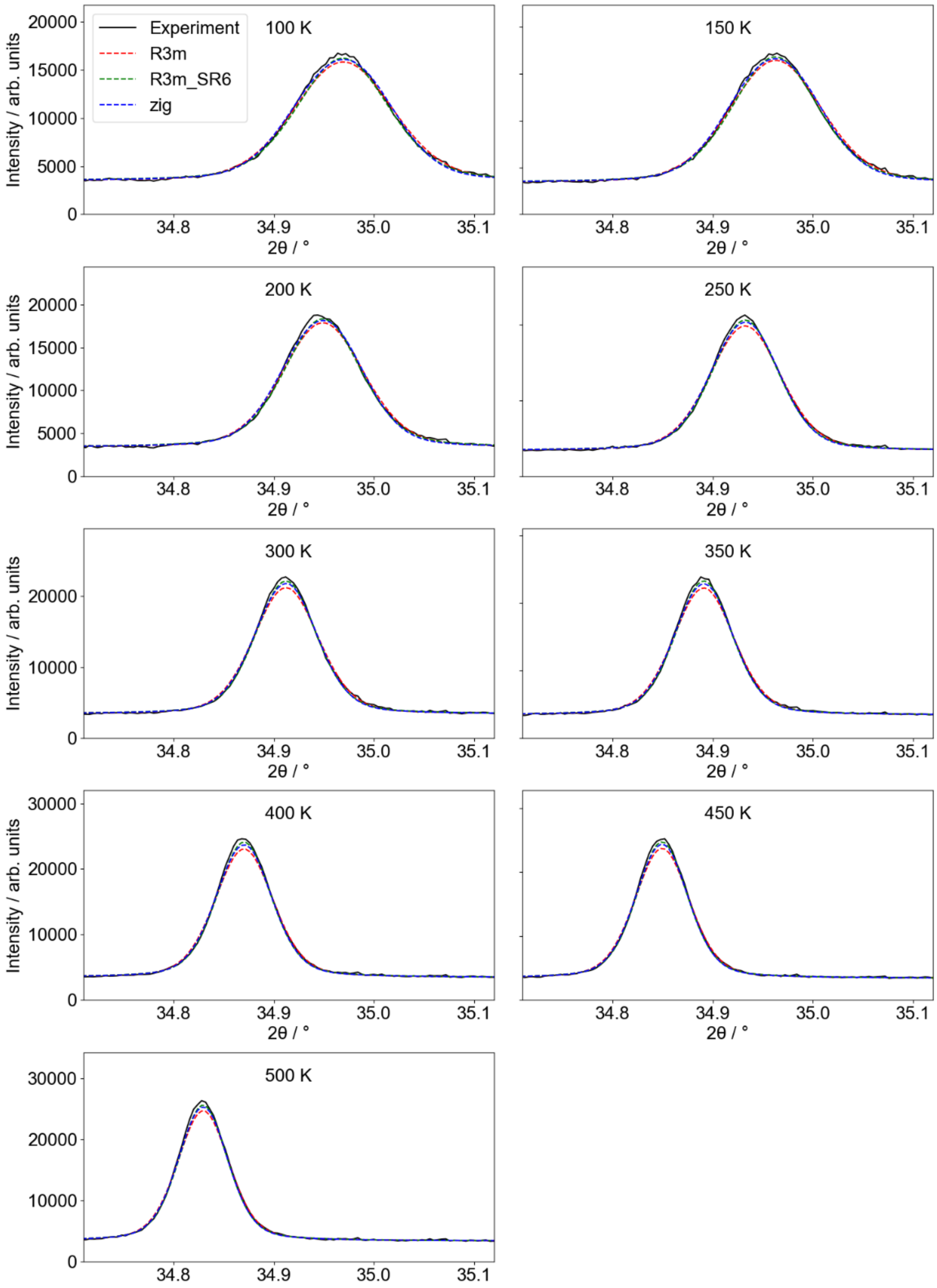

Figure S8. Comparison of the experimental (113) reflection (solid black line) with refinements based on a rhombohedral unit cell with isotropic broadening (red dashed line), rhombohedral unit cell with Stephens broadening (green dashed line), and monoclinic unit cell (blue dashed line).

(201)

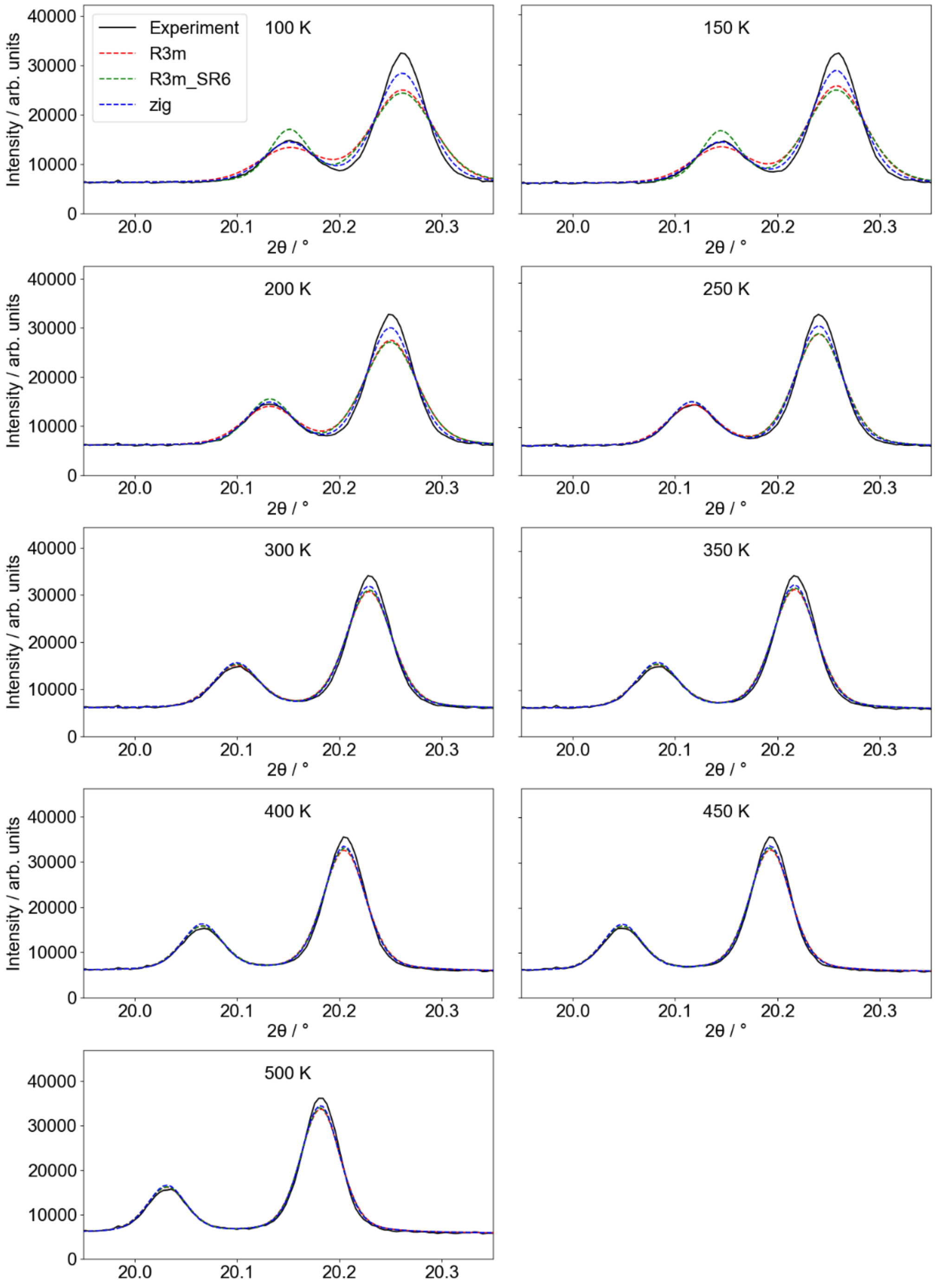

Figure S9. Comparison of the experimental (201) reflection (solid black line) with refinements based on a rhombohedral unit cell with isotropic broadening (red dashed line), rhombohedral unit cell with Stephens broadening (green dashed line), and monoclinic unit cell (blue dashed line).

(410)

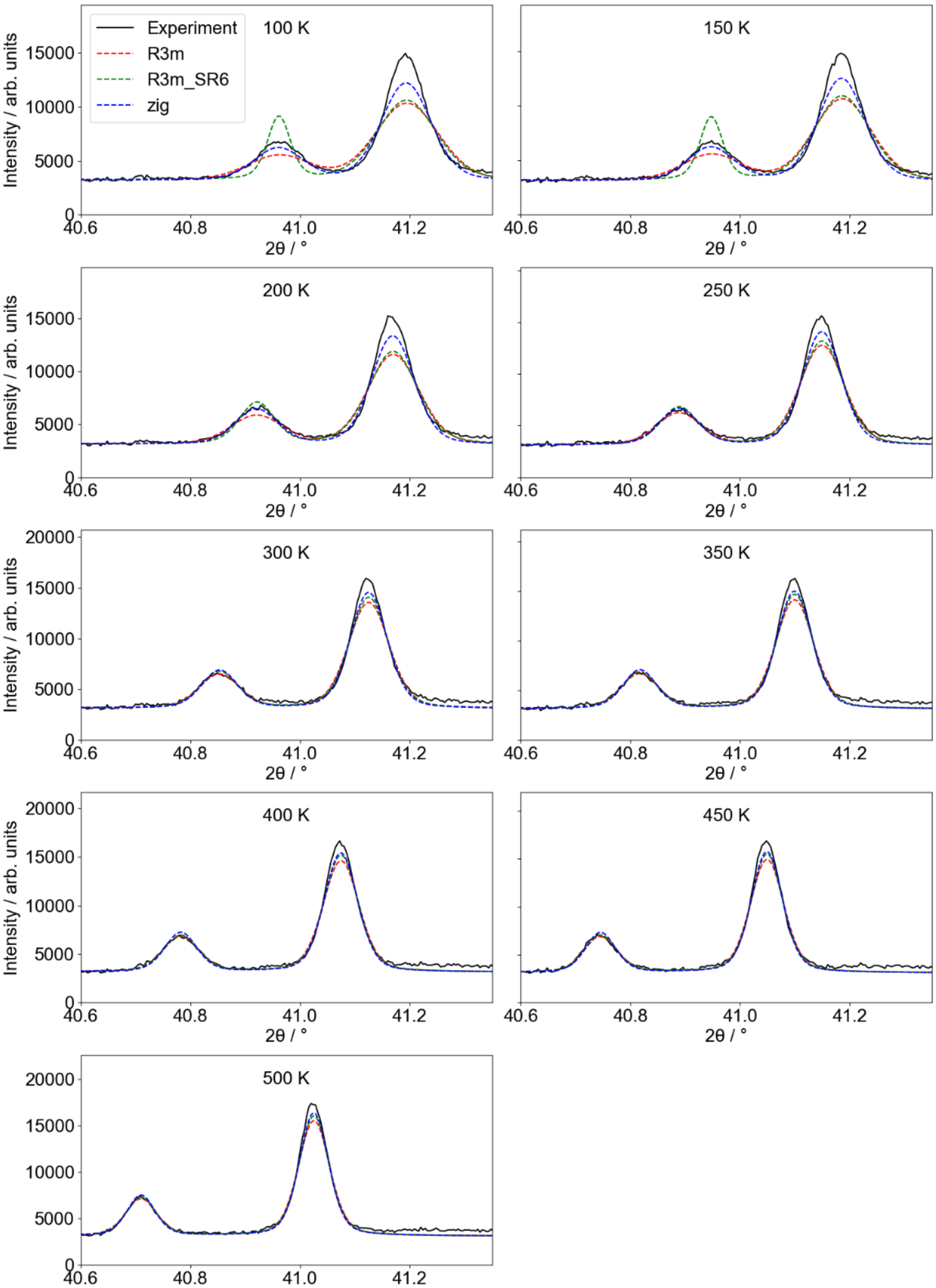

Figure S10. Comparison of the experimental (410) reflection (solid black line) with refinements based on a rhombohedral unit cell with isotropic broadening (red dashed line), rhombohedral unit cell with Stephens broadening (green dashed line), and monoclinic unit cell (blue dashed line).

Refinements based on symmetry-allowed distortions

Instead of refining the VT-X-ray diffractograms with a monoclinic structure, the diffractograms can also be refined based on symmetry allowed distortions of the parent rhombohedral structure. Figure 1a in the main text illustrates the symmetry relations between the two phases. The key difference between the structures is that a monoclinic distortion will break the trigonal rotational symmetry of the $R\bar{3}$m unit cell and thus allow Ni-O octahedra to adopt Jahn-Teller distortions. The lower symmetry of the monoclinic cell permits the oxygen positions to vary in the a, b, and c-directions compared to only in the c-direction for the $R\bar{3}$m structure. The Ni and Li positions are fixed to special sites.

The four unit cell parameters a, b, c and β are combined to describe four strain distortion modes. Two strain distortion modes – s1 and s2 in Figure S12 – retain the rhombohedral symmetry and are fully symmetric (irrep GM1+). These modes affect the rhombohedral lattice parameters a (mode s1 visualised in Figure S12) and c (mode s2). The other two strain modes break the rhombohedral symmetry (irrep GM3+) by changing predominantly the in-plane γ angle from 120° (mode s3) or by tilting the rhombohedral cell and changing the δ angle (mode s4).

The refined distortion modes as a function of temperature are shown in Figure S12. At low temperatures, γ differs from 120°, indicating a distortion from the rhombohedral structure. This distortion abruptly decreases in magnitude from 200 – 300 K. Above 300 K, the distortion is zero and γ remains constant at 120°, characteristic of the rhombohedral structure.

γ as calculated from the AIMD trajectories exhibits a similar trend, decreasing and showing an abrupt change in slope around 300 K (Figure S11). The overall change is somewhat larger than

in the experimental data which could be due to the size limitations of the simulation cell (fewer domains than in experiment).

The δ angle quantifies a tilt of the rhombohedral cell. The corresponding distortion s4 decreases with temperature, showing a distinct change in slope around *ca.* 260 K (Figure S12).

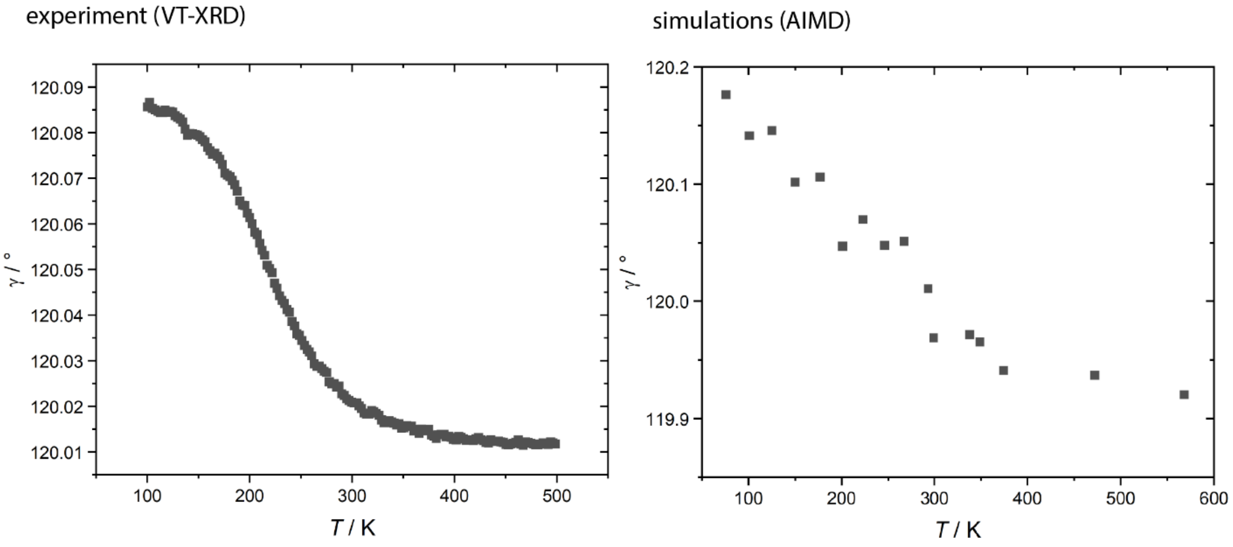

Figure S11. Comparison of γ as determined from VT-XRD and AIMD simulations. Both experiment and simulations show a decrease of γ with temperature and a transition to a constant value of *ca.* 120° around 300 K.

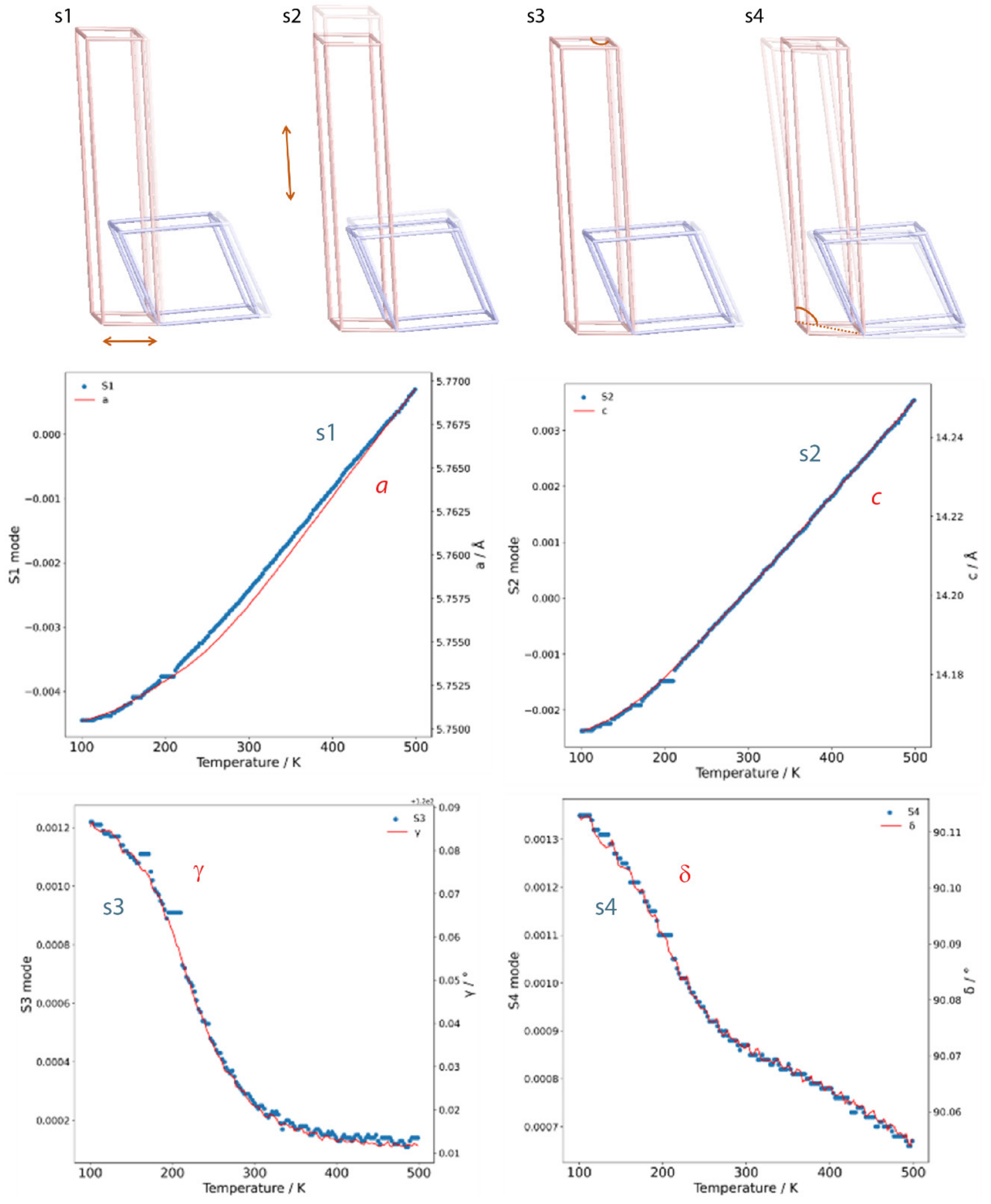

Figure S12. Strain distortion modes. The modes s1 and s2 affect the rhombohedral *a* and *c* lattice parameters and conserve symmetry. The s3 and s4 modes break the symmetry, changing the γ and δ angles, respectively.

Peak broadening on cooling

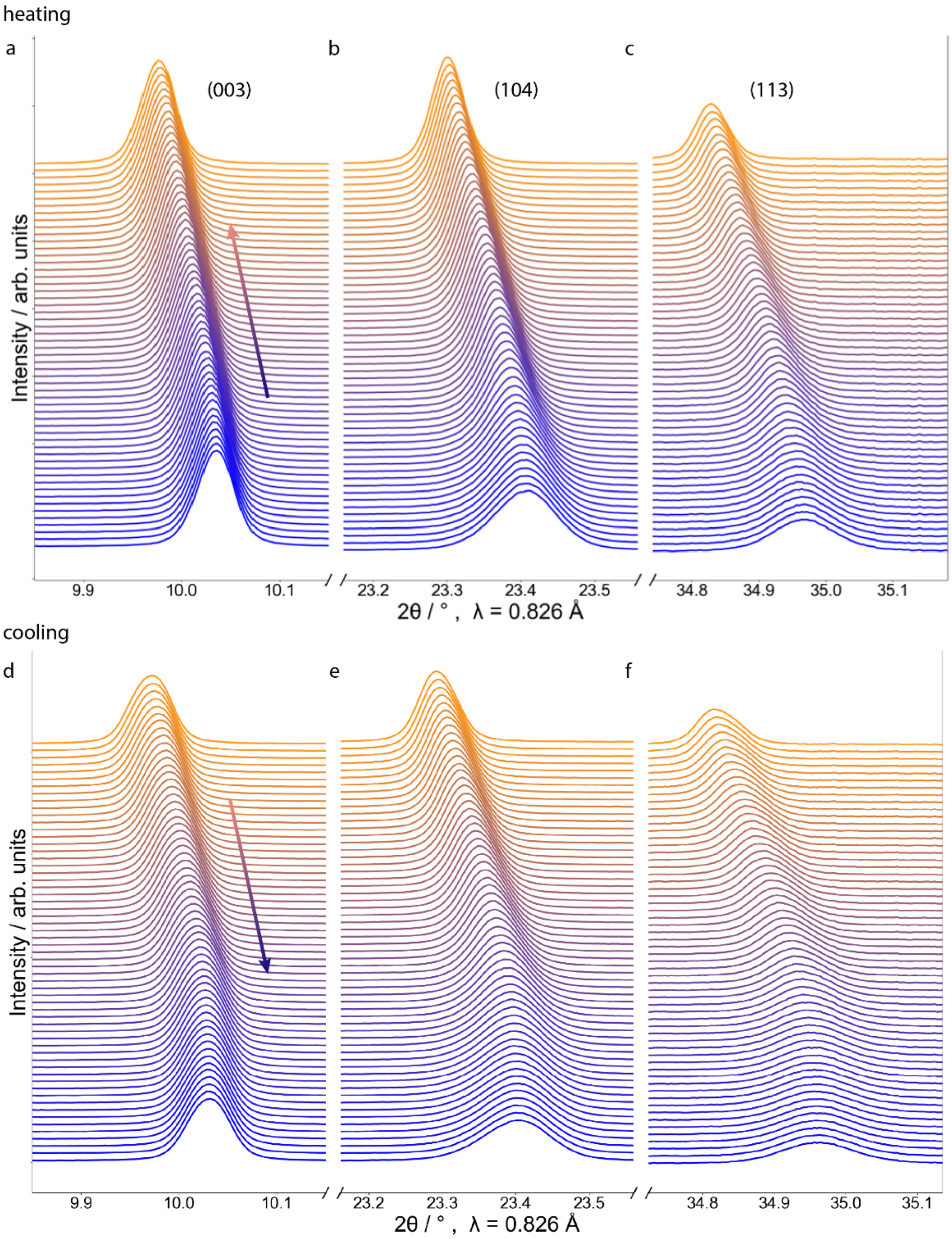

Figure S13. XRD peak widths as a function of temperature . Peak sharpening is observed on heating (top) and peak broadening on cooling (bottom) between 100 K (blue) and 500 K (orange) in steps of 7.5 K suggesting a reversible loss of monoclinic distortions on heating and reformation of distortions on cooling.
